# RBPSpot: Learning on appropriate contextual information for RBP binding sites discovery

**DOI:** 10.1016/j.isci.2021.103381

**Published:** 2021-10-30

**Authors:** Nitesh Kumar Sharma, Sagar Gupta, Ashwani Kumar, Prakash Kumar, Upendra Kumar Pradhan, Ravi Shankar

**Affiliations:** 1 Studio of Computational Biology & Bioinformatics (Biotech Division), The Himalayan Centre for High-throughput Computational Biology (HiCHiCoB, A BIC Supported by DBT, India), CSIR-Institute of Himalayan Bioresource Technology (CSIR-IHBT), Palampur, HP 176061, India; 2 Academy of Scientific and Innovative Research(AcSIR), Ghaziabad, Uttar Pradesh 201 002, India; 3 ICAR-Indian Agricultural Statistics Research Institute, Library Avenue, Pusa, New Delhi, Delhi 110012, India

**Keywords:** Sequence analysis, Systems biology, Omics

## Abstract

Identifying the factors determining the RBP-RNA interactions remains a big challenge. It involves sparse binding motifs and a suitable sequence context for binding. The present work describes an approach to detect RBP binding sites in RNAs using an ultra-fast inexact k-mers search for statistically significant seeds. The seeds work as an anchor to evaluate the context and binding potential using flanking region information while leveraging from Deep Feed-forward Neural Network. The developed models also received support from MD-simulation studies. The implemented software, RBPSpot, scored consistently high for all the performance metrics including average accuracy of ∼90% across a large number of validated datasets. It outperformed the compared tools, including some with much complex deep-learning models, during a comprehensive benchmarking process. RBPSpot can identify RBP binding sites in the human system and can also be used to develop new models, making it a valuable resource in the area of regulatory system studies.

## Highlights

• Efficient motif anchoring helps to get good quality contextual information on binding
• Realistic and high granularity datasets ensure better performance of the classifiers
• DNN models on the contextual features outperform more complex deep learning tools
• RBPSpot algorithm may be used to develop RBP binding models for other species also

## Graphical abstract

**Figure:**
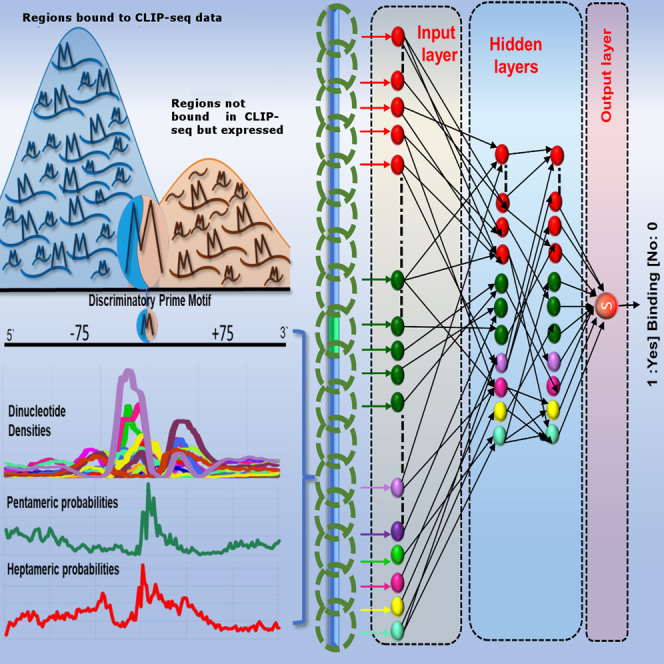

## Introduction

Advances in high-throughput techniques like CLIP-seq and Interactome Capture have drastically revised our understanding about RBPs which suggest that human systems are expected to have at least 1,500–2,000 genes coding for RBPs (Gerstberger et al., 2014; Castello et al., 2015). Unfortunately, we are still far behind in terms of information for these regulators as hardly ∼150 RBPs have been studied so far for their interactions with RNAs. There are certain limitations with these high-throughput experiments. These experiments are costly. They do not provide the entire RBP-RNA interactome spectrum, and at a time work for only one RBP in condition-specific manner. The CLIP-seq reads provide specific regions to look for interactions but do not provide the mechanistic details and explanations for the interactions. Using general motif discovery tools to identify the interaction spots have provided limited success in the case of RBPs as they either report too short motifs which have high chances of occurrences across the random data or they do not cover large spectra of instances. Unlike the transcription factors, RBP binding sites display sparse motif positional conservation. They are usually difficult to detect through such routine motif finding approaches. Besides the binding motifs, contextual sequence environment also guide the RBP-RNA interactions, adding further complexity to the process of discovery of the actual interaction spots. Therefore, this is an area which needs prime focus on deriving the principles of RBP-RNA interactions and their impact on regulation once we have enough CLIP-seq data. In one of the most remarkable works, RNAcompete, the authors identified *in-vivo* motifs for 207 different RBPs using pools of 30–41 bases long oligomers to which affinity of various RBPs was assessed for binding (Ray et al., 2013). RNAcompete also highlighted how conventional motif finding tools fail to discover the binding sites’ motifs for RBPs. At the computational front, some decent progress has been made to derive the models for interactions.

RNAcontext is among those pioneering tools which considered contextual information for RBP-RNA interaction discovery. It applied the structural preferences information for the RNAcompete motifs using the *ab-initio* RNA structure prediction tool, Sfold (Kazan et al., 2010). However, these *ab-initio* structural prediction methods’ reliability reduces with the length of RNA, making the structural information derived through them not reliable enough (Gardner and Giegerich, 2004). The next important stride came with probabilistic tools like RBPmap (Paz et al., 2014) and mCarts (Weyn-Vanhentenryck and Zhang 2016) which applied position-specific scoring matrices, supported motif clusters, HMM, and phylogenetic conservation to identify RBP RNA interaction spots. With GraphProt, a new generation of tools arrived which applied machine learning and leveraged new datasets developed from CLIP-seq experiments (Maticzka et al., 2014). It also applied the concept of differential RNA secondary structure information in a contextual manner to build the interaction models. A recently developed tool, beRBP, carried forward an approach similar to RBPmap when implementing the machine-learning method of Random-Forest while applying user provided motifs for primary screening (Yu et al., 2019).

With recent developments in the area of deep-learning, many deep-learning based RBP-RNA interaction detection approaches have been implemented recently. DeepBind deserves special mention among them because it pioneered a category where a robust general system was created to model nucleic acids and protein interactions using convolution neural network (CNN) (Alipanahi et al., 2015). Soon, DeepBind became a sort of prototype for almost all of the recent deep-learning based tools to identify the RBP-RNA interactions. Yet, the authors accepted that compared to transcription factors binding sites prediction performance of the deep-learning system, it was not up to the mark for RBPs. Taking the work further on deep-learning based RBP-RNA interaction detection, another prominent tool system, iDeeP, emerged which has come like a series of software like iDeep, iDeepS, and iDeepE (Pan and Shen 2017, 2018; Pan et al., 2018). These tools differ from each other for the way they apply various combinations of CNN and RNN/LSTM layers. A recently developed tool, DeepRiPe, has evolved a CNN- and GRU-based deep-learning approach while introducing a transcript’s region-specific information such as splice junctions (Ghanbari and Ohler 2020). DeepCLIP is another recently developed tool which detects RBP-RNA interaction spots while applying CNN in combination with bidirectional-LSTM and claims to detect sequence position-specific importance which could determine the contribution of various nucleotides in RBP binding (Grønning et al., 2020). These very recently developed deep-learning approaches have become much more complex than DeepBind, claiming much higher accuracy. Their complexity comes from adding complex layers above the regular dense hidden layers. These complex layers do the job of automatic feature extraction unlike the other machine-learning approaches where expert knowledge is applied to identify the important properties for suitable features’ extraction.

While reviewing these developments and tools, it looked imminent that there was an enormous scope for improvement in the approaches to find and locate RBP-RNA interaction spots. Some of the major points to consider would be as following: 1) The choice of datasets. A notable issue with all these algorithms is the choice of datasets, especially the negative datasets, which have mostly been too relaxed and unrealistic. Such datasets have made these tools prone to over-fitting and imbalance. They are either randomly shuffled sequences or regions randomly selected from those RNAs which did not bind the given RBP. 2) Motif searching approach. Most of the existing tools apply the motif finding approaches which were developed mainly for transcription factor–binding sites or protein motifs which are well conserved and continuous. Also, they begin with a predefined/user-defined motif or PWM derived from traditional motif-finding tools with user-defined length, which is not a natural approach, and one of the prime mistakes in the case of identifying the binding sites of RBPs as pointed out by the RNAContext study. RBP binding site motifs are usually sparse, discontinuous, and poorly conserved at many positions, which regular motif discovery tools may fail to capture sufficiently. 3) High dependence on *ab-initio* RNA structure prediction tools to derive the structural and accessibility information may be misleading. As already pointed out above, such tools do not provide correct information on actual complete RNA length. A better approach has been the consideration of dinucleotide densities for such purpose (Heikham and Shankar 2010; Černý et al., 2020). Consideration of RNA shape appears very much important as pointed out by DeepBind and some other recent works (Alipanahi et al., 2015; Jankowsky and Harris 2015; Gupta and Gribskov 2011). It has been reported that pentamers capture the essence of the nucleic acid’s shape accurately (Zhou et al., 2013), making them a suitable candidate to be evaluated along with dinucleotide densities to derive RNA structure and shape information. 4) Although the recent deep learning approaches claim good success through the automation of the process of feature extraction at the cost of added complexity, the effectiveness of such automated feature detection needs to be evaluated. Simpler models, if trained with carefully selected properties, are capable of outperforming complex models which typically work better under unstructured data conditions (Ryan 2021, https://towardsdatascience.com/the-unreasonable-ineffectiveness-of-deep-learning-on-tabular-data-fd784ea29c33).

Considering all these factors, here we present an efficient Deep Neural Net (DNN) based approach to build the mechanistic models of RBP-RNA interactions using high-throughput cross-linking data while considering data for 137 human RBPs from 99 experiments. An ultrafast k-mer spectrum search approach was applied to identify the most important seed regions in the sequence for contextual information derivation. Contextual information for 75 bases flanking regions around the identified seed-derived motif was extracted in the form of variable windowed position-specific dinucleotide, pentamers, and heptamers density-based propensities. The combined contextual information was provided to a two hidden layers–based deep feed-forward neural net to accurately identify the RBP binding spots in RNAs. The developed models were used to identify the interaction spots. They scored very high accuracy with remarkable balance between sensitivity and specificity as well as high performance consistency when tested across a large number of experimentally validated datasets. Molecular dynamics studies also supported these models. The developed approach has been implemented as a freely available web server and standalone software, RBPSpot. It was comprehensively benchmarked across three totally unbiased standardized datasets for performance measures along with five recently published tools for more than 50 RBPs, including more complex deep-learning–based tools. RBPSpot outperformed all of them consistently across all these datasets for most of the studied RBPs. Unlike most of the existing software which do not provide the option to build new models from the data, RBPSpot approach can be applied to detect human system RBP-RNA interactions with its inbuilt models and can be used to develop new models for other species and new RBP data also.

## Results and discussion

### Most of the RBP binding sites display a prime binding motif which covers a majority and exists along with co-occurring motifs

A total of 4,096 possible combinations of 6-mer seeds were individually searched through the mentioned inexact seed search approach in the peak data for every RBP. Most of these 6-mer seeds and their relatives were found occurring in at least 70% data with high statistical significance (p value<=0.01, binomial test). The number of most abundant 6-mer families varied across the RBPs [from RBM39 (3) to ELAVL1(17)]. The found significant spots for 6-mers worked as the seeds which were subjected to bidirectional expansion. After this step, only the prime motif candidates remained, which covered at least 70% of the CLIP-seq data and shared at least 70% similarity among them. After elongation, the best scoring expanded k-mer family for each RBP was considered as the prime motif in the RNA sequences interacting with the given RBP. It was found that at least one such prime motif existed for all the RBPs considered in this study, barring four RBPs. The size of the prime motifs varied from 6-10 bases for the RBPs. The most abundant prime motif was based on UCUGCAG for ALKBH5 (92.27%), whereas the least abundant prime motif was based on CCUGGAGG for SLBP protein (Table S1).

There were four different RBPs, *viz.* FXR1, SND1, ILF3, and U2AF1 which did not show any dominant seed k-mer occurring in at least 70% of the data. This suggested the possibility of multiple motifs working in a mutually exclusive manner. It was found that two different motif groups for these four RBPs were working almost in a mutually exclusive manner with small fractions of overlaps for some instances. The overlap levels between two such motif groups’ instances were 7.8% (FXR1), 6.8% (SND1), 8.25% (ILF3), and 9.5% (U2AF1) (Table S2). However, in these cases, the found 6-mers could not be expanded further as at least 10% of the data was lost because of this.

In this manner, the most significant motifs present in the cross-linking data of all these RBPs were discovered which could act as an anchor in contextual form. It was interesting to observe that the identified motifs could be clustered into various groups based on their similarity. Such observation is in concordance with previously conducted studies where RBPs were found sharing similar binding sites (Ray et al., 2013). This resulted in 28 clusters of RBPs with RBPs belonging to the same cluster while sharing a high level of similarity for their prime motifs (Figure S1).

**Figure 1:**
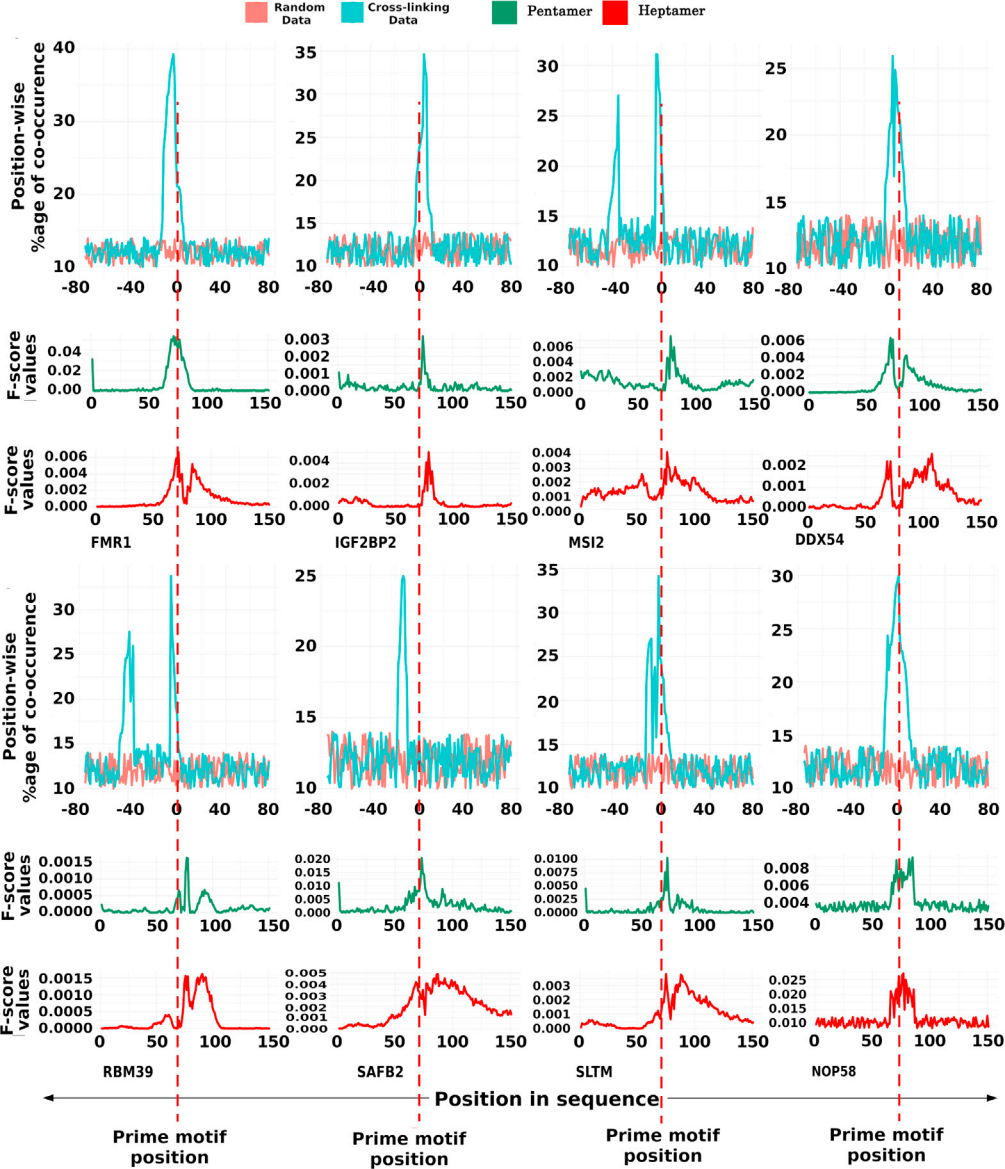
The co-occurring motifs positional preference The plots are showing the position specific existence of the co-occurring motifs with respect to the prime motif (coordinated at “0”). F-score values of other contextual features like position specific pentamers and heptamers distributions reflect this to the some extent. Most of these RBPs exhibited some secondary motifs which co-occurred with the prime motif in a position specific manner.

When these motifs were mapped back to the genome in order to derive the contextual information, several of them hinted at the coexistence of secondary supporting motifs for any given RBP. Such cases were studied further for co-occurrence of motifs where the most dominant motif would be supported by some other predominant secondary motif. All those sequences where the dominant motif existed were also searched for the supporting secondary motifs, where many of them were also experimentally reported previously. All co-occurring motif pairs possessed Jaccard similarity score <0.2, ensuring different motif partners being evaluated instead of the same motif repeating itself. Sequence regions where the motifs co-occurred displayed high statistical significance of co-occurrence rate for the motif pairs for the given distance (p value<<0.05; KS-test). For all the RBP models, a big difference was observed in the distribution of co-occurring motif pairs when compared to the random sequence regions, strongly supporting the existence of co-occurrence of motifs in RBP binding models of RNAs. Figure 1 illustrates some of these cases. In this manner, 178 statistically significant co-occurring motif pairs out of 297 motif pairs for 127 RBPs were obtained, strongly suggesting again that the context holds importance in RBP-RNA interactions. The details about co-occurring motifs are given in Table S3. Further, these motifs were also checked for frequency ratio >1 as discussed in the STAR methods section. All 178 statistically significant co-occurring motif pairs were found to have frequency ratio (FR) > 1. These co-occurring motifs were analyzed for the region flanking 75 bases from both sides of the prime motif. The reasons for considering this region become clearer in the following section.

**Figure 2:**
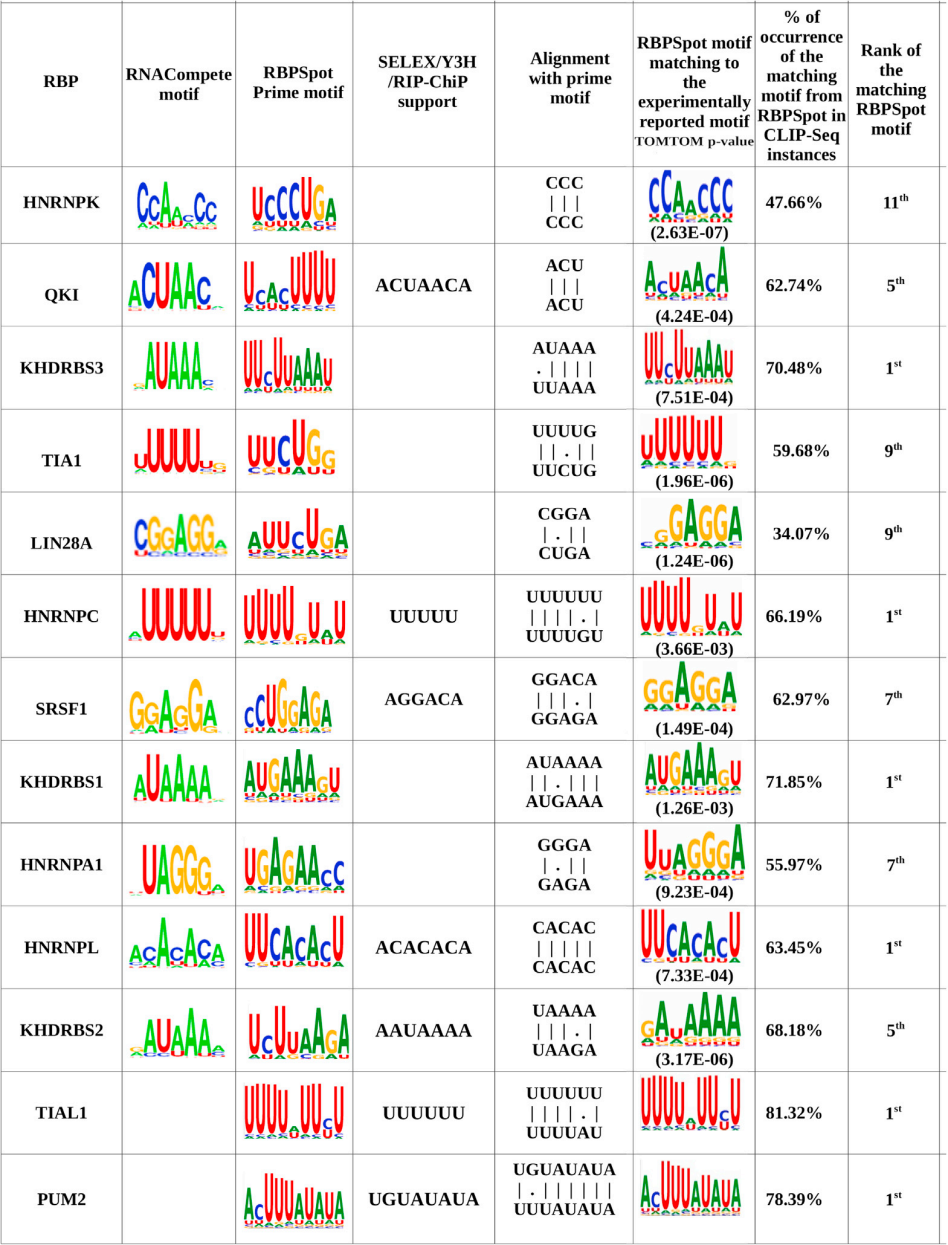
Comparison between experimentally reported motifs and motif identified in the present study Most of the previously reported motifs for the RBPs were detected by the approach presented in the current study. However, it was also observed that several of the previously reported motifs were not the prime motifs but comparatively cover lesser CLIP-seq data than the prime motifs identified in the present study. The last three columns show the matching motifs similar to the previously reported motifs, their status in CLIP-seq data coverage, and the corresponding motif’s rank.

The motifs reported in the present study were compared with the experimentally reported motifs. Many of the motifs found in this study matched with the experimentally reported motifs. However, it was also observed that several of these experimentally reported motifs were not the prime motif reported here but matched with other lower ranked motifs which either co-occurred with the prime motifs or were exclusively present, covering a comparatively lesser amount of CLIP-seq data than the prime motifs reported in the present study. Many of these non-prime motifs even matched with the experimentally reported motifs. It may be noted that the motifs reported through CLIP-seq were from much bigger data than experimentally reported motifs which may be a subset of a larger dataset, influencing their relative ranking. Their occurrence in the cross-linking data varied from 34.07% to 81.32% while the prime motifs reported in this study mostly covered at least 70% of CLIP-seq data. Figure 2 provides a snapshot of the comparison between experimentally reported motifs with motifs identified in the present study. The observed percentage of the matching motif reported in the figure was calculated over the CLIP-seq instances specific to the corresponding RBP only because the CLIP-seq experiments are always RBP-specific. Most of the time the secondary but experimentally matching motifs co-occurred with the prime motifs. For several RBPs, their occurrence percentage went very close to the value reported for the prime motif in a mutually exclusive/independent manner.

### Contextual information surrounding the anchored motif is critical for RBP binding sites recognition

**Figure 3:**
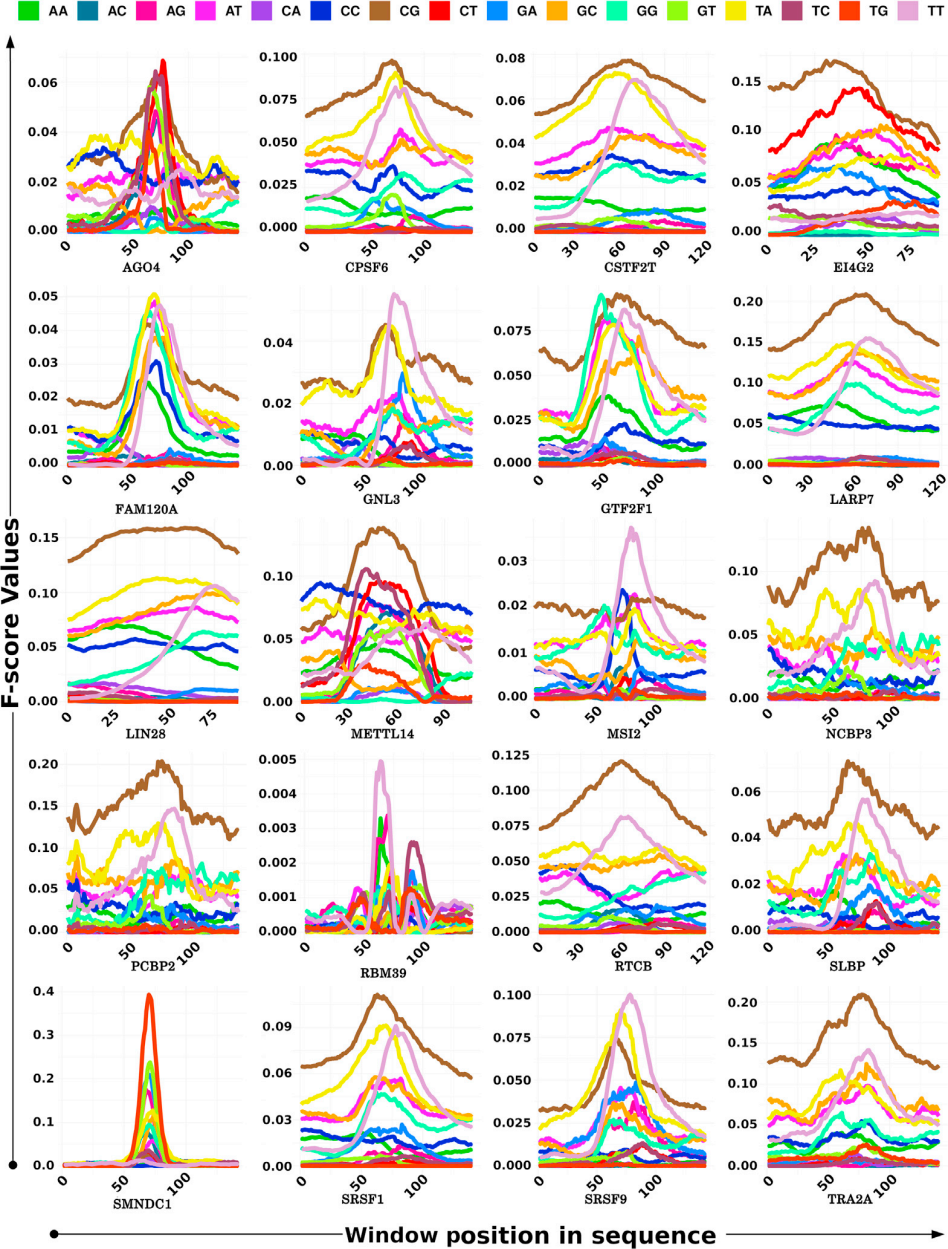
F-score distribution of dinucleotide densities at different positional windows for the target regions and their flanks for Cluster#1 members Context specific dinucleotide density distribution emerged among the most important features for all the RBPs taken in this study. Their densities worked as important features at variable window sizes and distances for different RBPs. Here, Cluster# 1 members’ data is shown. They shared high similarity among themselves for their prime binding motifs, but their contextual information and density profiles differed a lot. An enriched contextual “CG” distribution of these regions was found consistently as a distinguished property for the regions binding the RBPs.

The motif discovery and anchoring steps helped in selecting the more appropriate contextual information and features. The discovered motifs above worked as a point to zero upon to consider the potentially significant interaction spots across the RNA. However, such motifs alone may not hold much higher stake than that as they may appear in the non-binding regions also despite being statistically significant for the binding regions. Evaluation of the context for their functional role thus becomes essential. In this regard, 75 bases from both flanking regions were considered where the motif region worked as the anchor. Previously, it has been found that ∼75 bases of flanking regions around the potential interaction sites in RNAs capture the local environment for structural and contributory information effectively (Heikham and Shankar 2010). Also, the RBPs which interact with the RNA through multiple domains use multiple interaction sites which are usually concentrated around a local region instead of being long-distanced interaction spots. The contextual information came in the form of other co-occurring motifs, sequence specific information, position specific information, and structural/shape information which could exhibit sharp discrimination between negative and positive instances. The contextual information was derived from the features based on four major properties: (1) 7-mers distribution probabilities for each position; (2) 5-mers distribution probabilities for each position; (3) dinucleotide densities for each positional window; and (4) structural triplet densities covering 27 combinations of structure triplets arising from the dot-bracket structural representation from RNAfold predicted RNA structures. Various features generated based on the above-mentioned properties were evaluated for their discrimination potential between the positive and negative instances. The most important top 100 features are given in Table S4. Among them, the features originating from the position-specific dinculeotide densities appeared as the most important one among the top features. Some pentamer and heptamer features were also present among these top features. Dinucleotides densities reflect the structural and accessibility properties of the nucleic acids, as mentioned above. A very striking observation was also made here. Most of the positive instances of flanking regions displayed enrichment of CG. Approximately 69% of RBPs’ target regions exhibited CG among the most dominant features for each position, whereas for the rest of the RBPs, UU and UA were among the most prominent features. Besides this, it was also observed that RBPs which shared high similarity for their binding motifs and which belonged to the same cluster (Figure S1) differed substantially for this contextual information. Their flanking regions displayed different distribution patterns of these features. Figure 3 presents an example of one such group, RBPs belonging to AGO4 cluster (Cluster 1). As can be noted in this figure also, CG is remarkably enriched for the binding site regions. Therefore, despite having very similar binding site motifs, they differ in their binding which is influenced by context. Also, the universal prominence of CG in the RBP binding regions reinforces the theory which suggests their regulatory roles in stationing the binding factors and supporting the binding motifs (Hartl et al., 2019). They may also be studied further for RNA modifications which are considered critical for RBP binding dynamics.

For 12 RBPs, pentamers were also found in the top 20 features for different positions whereas heptamers were found for 10 RBPs in the top 20 features. Among the top 100 features, almost in 90% of the cases, heptamers and pentamers marked their presence. Significant difference was observed between the positive and negative instances with respect to the F-score for positions. This also suggested that a substantial amount of information was being held by the flanking regions around the binding motif which may be one of the determinants for contextual interactions between RBP and RNA. A series of t-tests between the positive and negative instances for the various features also supported this. Biologically, heptamers and pentamers were expected to reflect any supporting co-occurring motifs near the prime anchored motif. However, the pentamers were considered specifically to capture the shape properties which have been called important for protein and nucleic acids interactions, more so for the cases where sequence motifs are not clear or prime (Alipanahi et al., 2015; Zhou et al., 2013). A closer look with these pentamers and heptamers revealed that for many RBPs binding sites the pentamers were prominent in the flanking regions where the co-occurring secondary motifs existed (Figure 1). Although heptamers were found more reflective of this phenomenon, the impact of each of these properties on the discrimination capacity between true binding sites and negative sites was also clear when evaluated directly on the machine learning models for performance, as transpires in the following section.

### DNN implementation of the RBP binding site models consistently achieved high accuracy

Before combining the features to build the collective models for RBP-RNA interactions, one more assessment of contributions by the above-mentioned properties in discrimination was done. Classification assessment was done for each given property separately before joining them together while using XGBoosting. This was done to get the preliminary idea about the individual contribution made by each of the contextual properties toward an accurate classification.

**Figure 4:**
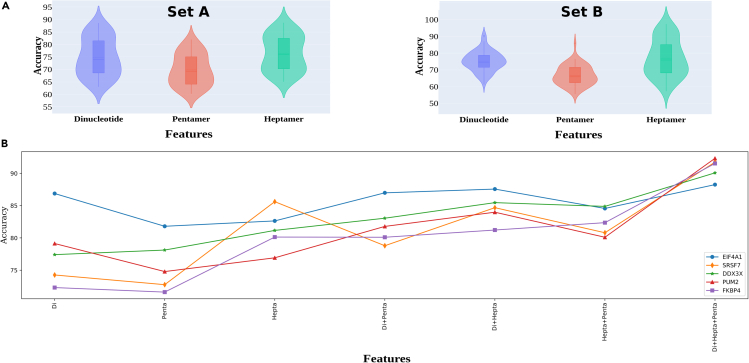
Assessment of the three main properties in discriminating between the negative and positive instance (A) Violin plot distribution of the accuracy when dinucleotides, pentamers and heptamers densities were used separately for Set A and Set B RBPs. (B) Impact of the combination of the dinucleotide, pentamers, and heptamers properties based features. These features appeared highly additive and complementary to each other as the performance in accurately identifying the binding regions increased substantially when they were combined.

For the pentamers-based classification, the accuracy varied from 60.23% (U2AF2) to 82.01% (FKBP4) for Set A RBPs with an average of 69.8% accuracy. For the heptamers, it varied from 65.01% (FXR1) to 88.72% (FXR2) with an average accuracy of 76.49% for Set A RBPs. Similarly, for Set B RBPs, the pentamers accuracy varied from 55.6% (DHX9) to 86% (EIF4A1) with an average of 66.7% accuracy. For the heptamers, this value varied from 57.23% (MOV10) to 97.47% (EIF4A1) with an average accuracy of 77% for Set B RBPs. For the structure triplets of RNA we used different window sizes. However, none of the windows achieved more than 63.39% accuracy for any RBP, clearly supporting the earlier observation that *ab-initio* structure prediction derived features do not add much value due to their innate limitations. Therefore, this feature was not further taken for the final model building. Accuracy for dinucleotide densities based classification varied from 63.04% (FMR1) at 43 bases window size to 88.6% (RBFOX2) at 71 bases window size with an average accuracy of 75% for Set A RBPs. Similarly, for set B RBPs, the accuracy of dinucleotide density based classification varied from 61.46% (DHX9) at 71 bases window size to 90.57% (EIF4A1) at 91 bases window size with an average accuracy of 75.25% (Tables S5 and S6). The results here displayed concordance with the observation made in the previous section where importance of the contextual dinucleotide density information based features emerged among the most important ones for the detection of binding sites. Figure 4A presents the violin plots for accuracy score distributions observed for the classifications done by each of these properties for all the studied RBPs.

After getting optimum window size for dinucleotide densities, we combined these three features (pentamers probabilities, heptamers probabilities, and dinucleotide densities) to build the final models. The final models were built using XGBoosting and DNN. The reason for considering these two different approaches was that: 1) they reflect two different learning approaches, Shallow and Deep; 2) they complement each other as XGBoost works well for the cases with comparatively lower training data while DNN performance is good where the learning data is higher; 3) both approaches work very well for the conditions where the dimensions are high, as was with this study. Combining the features based on the above-mentioned properties was done in a gradual manner in order to see their additive effect on the classification performance. As it is apparent from Figure 4B, which showcases the DNN classifier’s performance for five RBPs, the performance of the classifiers kept increasing upon addition of more features, where consistency also increased as can be seen through the bandwidth of the plots for the five RBPs. In addition, the dinucleotides based contextual features emerged most critical as the biggest leap in the performance was noted when it joined the heptamers and pentamers based features. Any pairing of these three properties features gave almost similar performance, but the sharpest rise was observed in performance when the contextual dinucleotide information based features were added to the pentameric and heptameric features. Besides this assessment of the impact of the mentioned features, one more interesting test was conducted across a set of randomly selected 50 RBPs to assess the impact of the identified prime motifs on the observed performance. The DNN models for them were rebuilt without the prime motif information with random placement of the central window, which was earlier anchored by the prime motif, in the CLIP-seq data for the positive dataset. In this manner, the prime motif’s contribution was removed. The developed models observed a drastic drop in their average performance by ∼15%. Thus, the prime motif emerged as the most influential feature of the DNN’s performance as it was also responsible for extracting the right contextual information for other considered features.

After combining the features, there were a total of 1,198 features for individual RBPs (ZNF184) to 2,544 (EIF4A3, EIF4G1, EWSR1, HNRNPD, HNRNPL, KHDRBS3, NOP58, etc.). The feature numbers varied due to the different sizes of the best performing dinucleotide densities windows. These features were used in XGBoost machine learning where the average accuracy of 85.07% (Avg F1-Score: 84.64%, Avg MCC:0.79) was obtained and where the accuracy values varied from 79.19% (FXR2) to 90.81% (RBM47) for Set A RBPs. It was found that the average accuracy of 84.08% (Avg F1-Score:82.66%, MCC: 0.69) was obtained for Set B RBPs, where the accuracy values varied from 66.34% (MOV10) to 96.78% (EIF4A1). The same set of the combined features was also used in the DNN implementation. The DNN works better with higher dimensions and instances to learn from. In the input layer the combined features were used where two hidden layers gave the best performance and the number of nodes per hidden layer varied from 700 to 1,300. Details of the implementation are already given in the STAR methods section. The DNN achieved an average accuracy of 92.25% (Avg F1-Score: 91.97%, MCC: 0.85) for Set A RBPs which was much higher than XGBoost. For Set B RBPs, an average accuracy of 83.47% (Avg F1-Score: 83.18%, Avg MCC: 0.67) was achieved by the DNN models, which was slightly lower than XGBoost. Complete performance details can be found in Tables S5 and S6.

In general, it was apparent that the DNN approach was sensitive toward the volume of training instances as it was found to perform better where the number of instances were higher. But the biggest impact on performance was observed on the granularity of dataset creation. Performance of DNN was especially more marked here, as was evident from its performance plot on Set A datasets. On Set A, the DNN models performance hardly fell below 90% accuracy. Even XGBoost’s performance was better with Set A as compared to Set B. It needs to be recalled that Set B was made for those RBPs whose RNA-seq data was not available for the considered CLIP-seq conditions. In such scenarios, those RNAs were considered to generate the negative instances whose some regions were present in the CLIP-seq data suggesting their expression. From the same RNA, those regions were selected which had the prime motifs but were not binding to the RBPs, were not reflected in the CLIP-seq data, and were distant from such binding regions. While Set A negative instances were clearly those regions which were expressed during the CLIP-seq experimental condition and possessed the prime motif, no region of the RNA itself bound to the RBP. Thus, although the overall performance with Set B was still good and better than the datasets used by the compared tools, as also transpires in the next section, at the same time this also reflects the importance of having a refined granular dataset like Set A.

**Figure 5:**
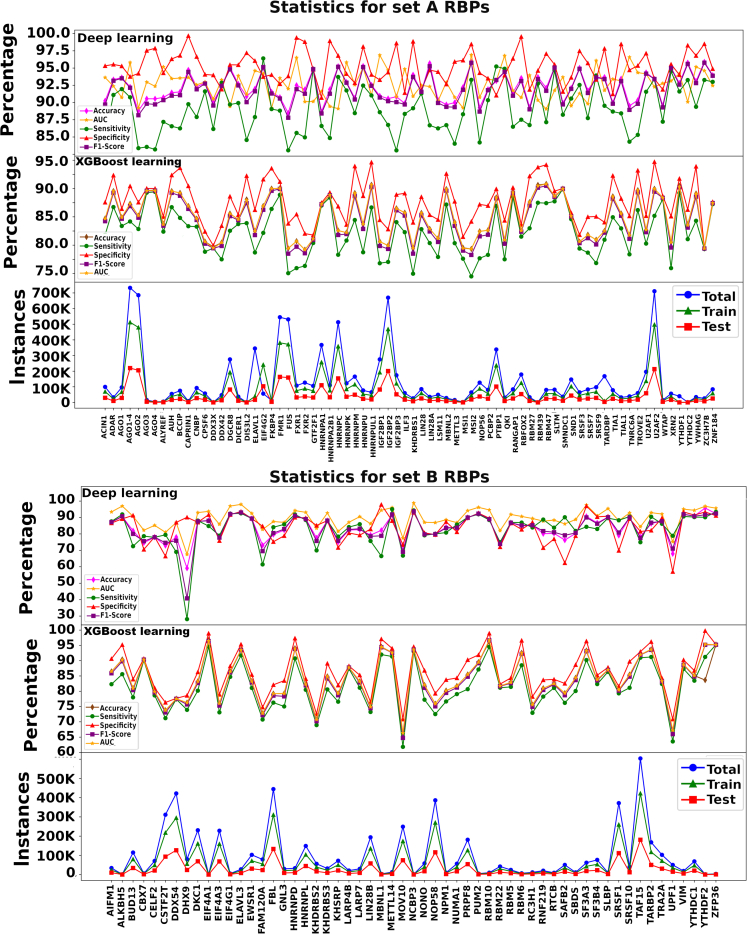
Performance metrics for RBPSpot (A) The first plot is showing the accuracy, AUC, sensitivity, specificity, and F1 scores for the DNN models for Set A RBPs. The second plot is showing the same metrics for the gradient boosting method. The third plot is showing the corresponding number of instances in the test, train, and in the total data for Set A RBPs. (B) The first plot is showing the accuracy, AUC, sensitivity, specificity, and F1 score for the deep learning models for Set B RBPs. The second plot is showing the same metrics’ values for the gradient boosting method with Set B RBPs. The third plot is showing the number of instances in the test, train, and in the total data for Set B RBPs. RBPSpot scored the highest for all the performance metrics with remarkably consistent performance across a large number of RBPs and the datasets.

**Figure 6:**
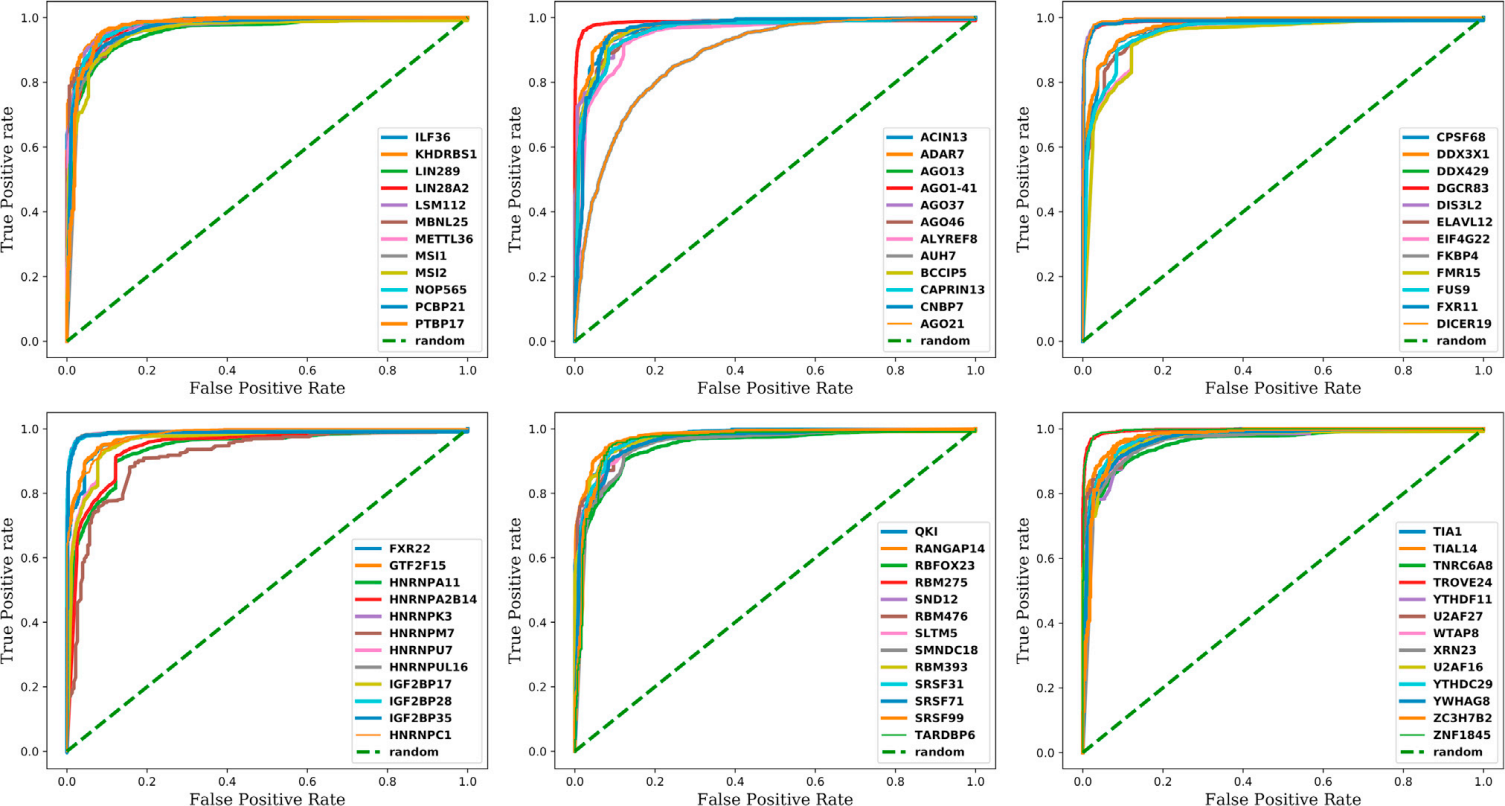
AUC/ROC plots for Set ARBPs The AUC/ROC plots for the deep-learning models for some of the RBPs clearly showcase the robustness and highly reliable performance of the implemented DNN models.

As evident from the considered performance metrics for various RBPs (Figure 5) and the AUC/ROC plots (Figure 6), the developed approach in this study, named RBPSpot, showcased a consistently high and reliable performance for most of the studied RBPs. It also provides the largest number of models for RBPs binding developed from CLIP-seq data to this date.

### Comparative benchmarking: RBPSpot consistently outperforms all the compared tools

A very comprehensive benchmarking study was performed where RBPSpot was compared with five different tools, representing different approaches of RBP RNA interaction detection: RBPmap (probabilistic approach), beRBP (a Random Forest machine learning based approach claiming the highest accuracy in its category), DeepBind (the first deep-learning based approach), iDeepE, and DeepCLIP (representing some very recent and more complex deep-learning based tools). Besides this, the benchmarking has also considered three different datasets to carry out a fully unbiased assessment of the performance of these tools across the different datasets. The first dataset considered in the benchmarking study was derived from the RBPSpot dataset. The second dataset considered in the study was the one evolved during development of the GraphProt software which has been used largely by various other datasets for model building and performance benchmarking purposes. The third dataset used in this benchmarking study was the one used by beRBP software which too has been used by many other tools for the same purpose. Details about these datasets have already been discussed above and in the STAR method section.

**Figure 7:**
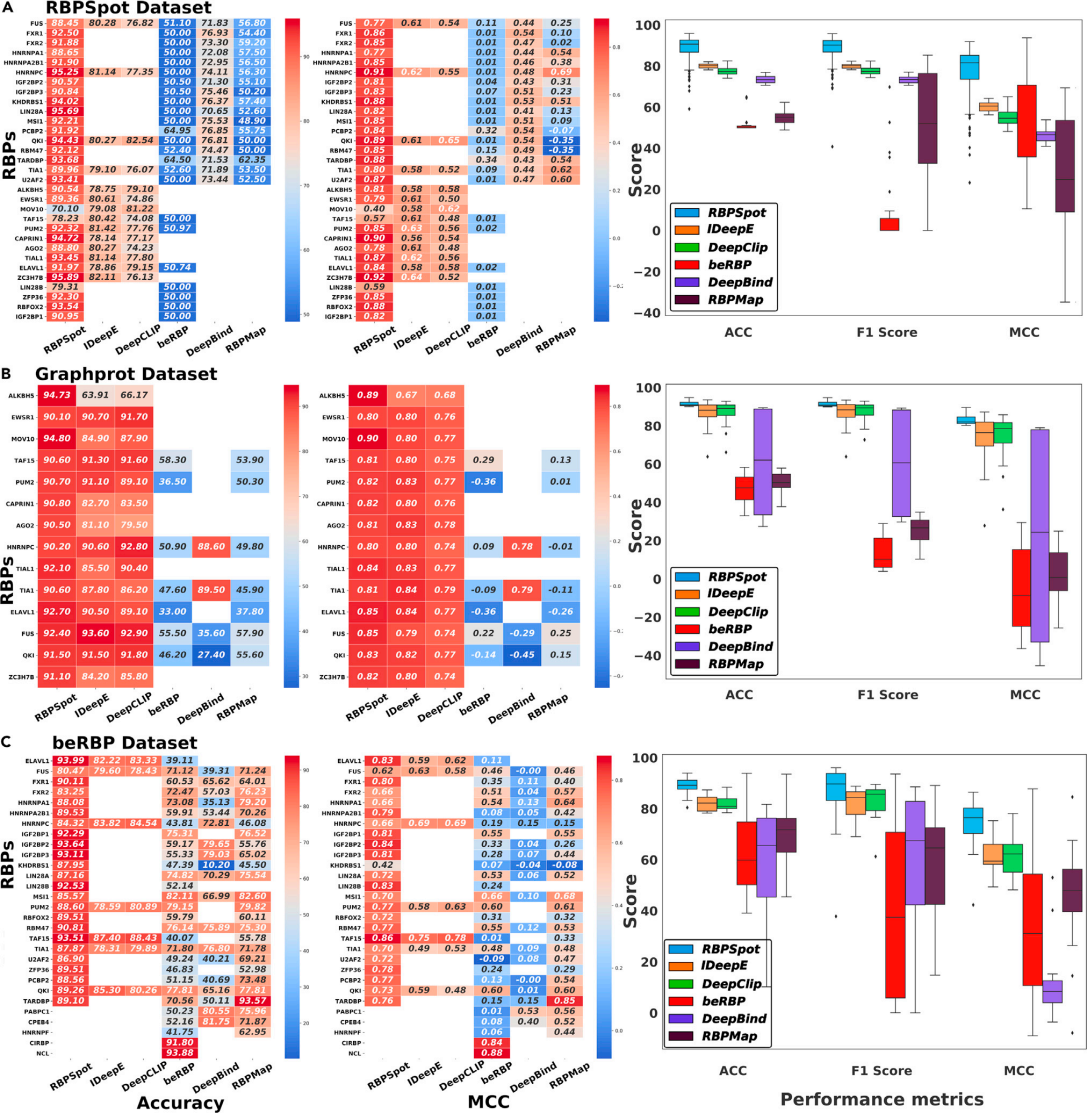
Comparative bench-marking results for RBPSpot when compared to beRBP, DeepBind, RBPmap, iDeepE, and DeepCLIP for the three different datasets (A–C) The bechmarking result for RBPSpot dataset, (B) Graphprot dataset, and (C) beRBP dataset. For each of these datasets, the performances were evaluated for the various performance metrics. The heatmaps are for accuracy and MCC values for each dataset for some of the evaluated RBPs. The last plot is the box plot which provides the snapshot of the distribution of these metrics scores for the studied RBPs. From the plots it is clearly visible that for all these datasets and for almost all of the RBPs, RBPSpot consistently outperformed the compared tools for all the metrics. The box plot suggests that RBPSpot not only performed best overall but also the dispersion of its various metric scores were much less than the other compared tools. Some of these tools exhibited enormous variation in the distribution of their metric score suggesting their unstable performance. Complete details of all RBPs including the corresponding average performance heatmaps and radar plots are given in the associated Tables S10, S11, and S12, Figure S2.

All these six software were tested across these three datasets for more than 50 RBPs where RBPSpot outperformed all of them across all the datasets, for all the performance metrics considered (Figures 7 and S2). RBPSpot scored an average accuracy of 88.43% and an average MCC value of 0.77 on RBPSpot dataset. It had an average accuracy of 91.63% and an average MCC value of 0.83 on GraphProt dataset, and an average accuracy of 88.9% and an average MCC value of 0.74 on beRBP dataset. Among all the considered performance metrics, MCC stands as the most important one as it gives a high score only when a software scores high on all the four performance parameters (true positive, false positive, true negative, false negative). A good MCC score signifies the robustness of the model and its performance consistency. RBPSpot emerged as the most robust algorithm among all these compared software with very high consistency of performance (t-test p values: 4.03E-07 (vs iDeepE), 9.79E-08 (vs DeepCLIP), 2.01E-43 (vs beRBP), 6.34E-32, 1.45E-37 (vs DeepBind)). As it is visible from the score distribution for all the metrics, RBPSpot also exhibited the least dispersion of scores for all the studied RBPs and for all the three datasets, confirming the precise performance achieved by RBPSpot compared to other tools. RBPSpot’s performance also points out that more appropriate features may be learned through training on biologically relevant properties to derive better discrimination power using machine learning approaches which may be amalgamated with Deep Neural Nets with much less complexity and superior performance than applying complex deep-learning layers which automate the feature extraction step. The problems where features are identifiable and can be structured, the simpler machine learning models may outperform the complex deep-learning approaches in such scenario.

**Figure 8:**
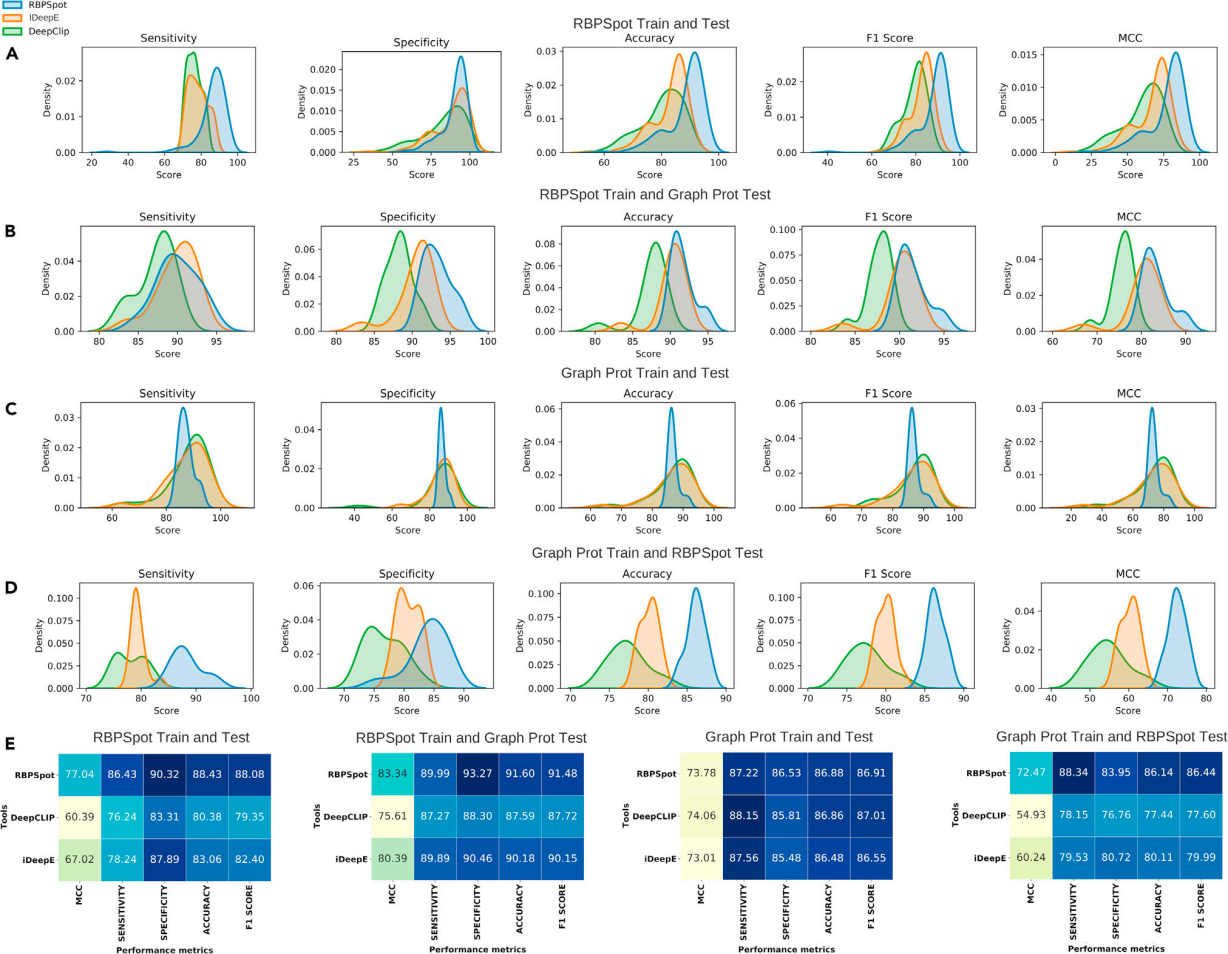
Performance benchmarking with different combinations of train and test datasets In this part of performance benchmarking the impact of datasets was also evaluated. Since this part required rebuilding of RBP-RNA interaction models from scratch and from the provided user-defined data, only two tools other than RBPSpot qualified for this criteria (DeepCLIP and iDeepE). These tools provide the capability to build new models from user given datasets. These tools were originally developed on GraphProt dataset. Therefore, in this part of benchmarking RBPSpot and GraphProt datasets were considered and 4 different train-test datasets combinations were studied. (A–E) Distributions for various performance metrics for the compared tools and the corresponding datasets have been given as Kernel density plots: (A) RBPSpot train and test, (B) RBPSpot train and GraphProt test, (C) GraphProt train and test, and (D) GraphProt train and RBPSpot test. For every such combination, the average performance metrics scores are given in the form of heatmap (E). The plots clearly underline that RBPSpot consistently outperforms the other two tools for all the metrics on all these different combinations of train and test datasets, where again the consistent and precise performance of RBPSpot was an important observation. Consistently high MCC scoring by RBPSpot underlined it as a robust and balanced algorithm where dispersion in performance metrics was minimal. Also, the performance of all the compared tools improved when the RBPSpot dataset was used for training, clearly suggesting the importance of having a refined dataset. The RBPSpot dataset presented here emerged as a better dataset for such studies.

The above-mentioned benchmarking was done for all the tools while keeping their original training dataset and models for RBPs. Most of the existing tools do not provide the option to build user-specified models of RBPs using their algorithms but come with their own pre-built models. This limits the scope to test the algorithms with different combinations of train and test datasets. Yet, the two best performing tools after RBPSpot, iDeepE and DeepCLIP, provided the scope where the users may build their new models with their own datasets. Figure 8 presents the results for this part of benchmarking where RBP models were rebuilt and tested using the four different combinations of training and testing datasets. RBPSpot outperformed the compared software, iDeepE and DeepCLIP, for all the combinations of datasets, for all the considered performance metrics. Like the previous benchmarking study, here too, RBPSpot scored the highest among all the software for all the combinations of datasets with a remarkable consistency (t-test p values: 1.78E-27 (vs iDeepE); 2.31E-35 (vs DeepCLIP)). As transpires from the kernel density plots in Figure 8, RBPSpot maintained its least variability and dispersion of performance scores and continued to display a strong balance in detecting the positive and negative instances with high and similar level of precision. This was reflected by high scoring on all the four parameters of performance resulting in consistently high MCC values, which confirmed the robustness of the algorithm. Also, it was observed that the performance of all the compared software was better when RBPSpot dataset was used for training. The original implementations of iDeepE and DeepCLIP have used GraphProt dataset. Both these software performed better when their original dataset for model building was replaced with RBPSpot training dataset, underscoring better and more realistic composition of RBPSpot dataset. RBPSpot consistently scored highly across all the comparative tests and clearly outperformed the compared tools. The complete details and data for the benchmarking studies are given in Tables S7, S8, S9, S10, S11, and S12

Anchoring based on the identified prime motifs followed by the contextual features around the prime motifs based on dinucleotide profiles in a positional manner, heptameric profiles, and pentameric profiles contributed the most to the observed better performance of RBPSpot. To this, the choice of DNN over shallow machine learning approach further helped to enhance the performance while keeping the architecture simpler and more efficient than the compared deep-learning approaches. In terms of computational efficiency, RBPSpot was found to be performing at least ∼5X times faster than iDeepE and ∼8X times faster than DeepCLIP on any given machine.

### Structural and molecular dynamics analysis supports the RBP binding site models

**Figure 9:**
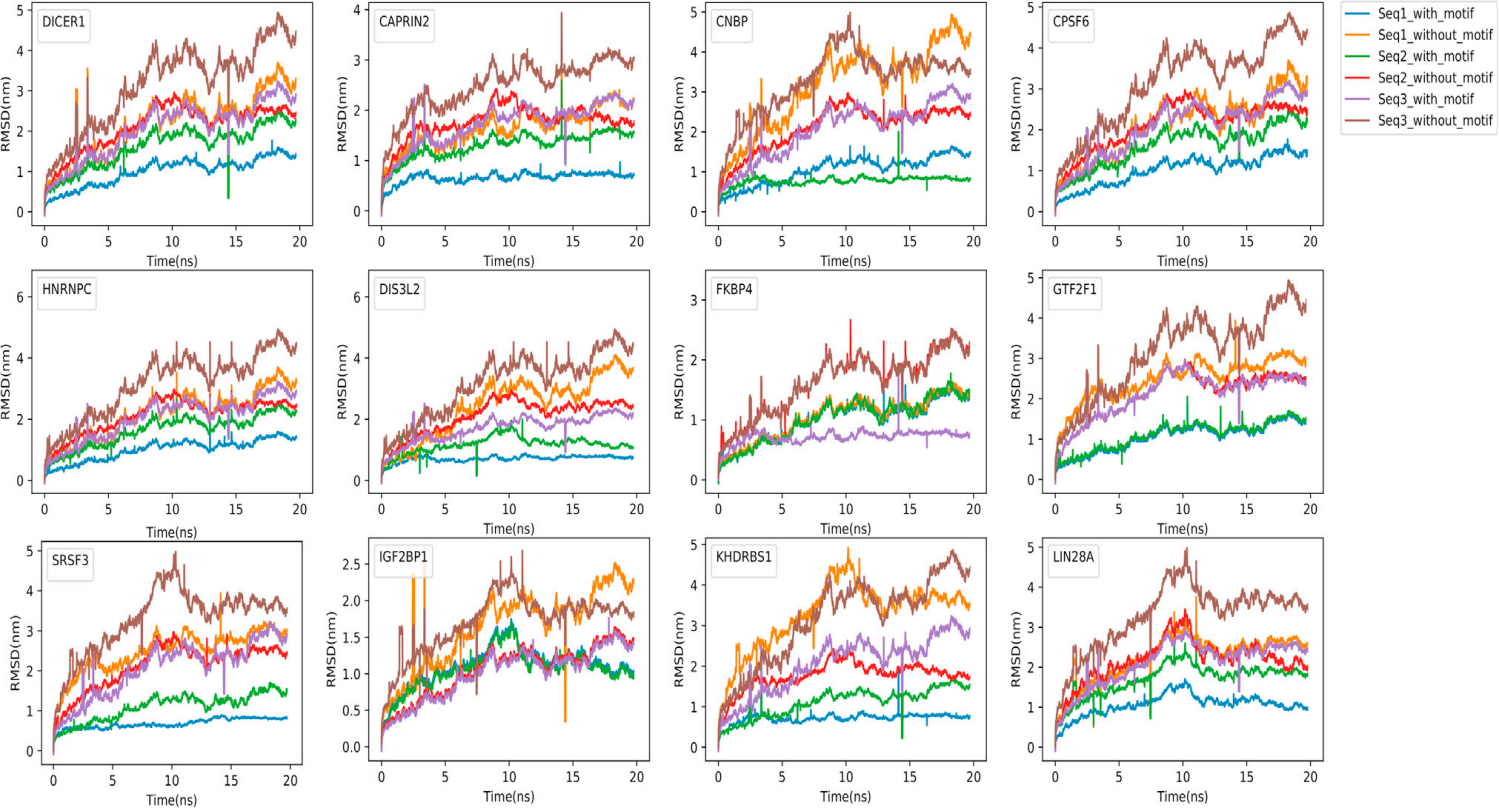
Comparative time-dependent root mean square deviations (RMSD) plots for 12 different RBP-RNA complexes (with and without the prime motif) The trajectory was measured at 300 K for the 20-ns. The trajectory arcs for RBP-complex of three randomly selected RNA sequences with motifs are shown in blue, green, and violet spike arcs whereas the trajectory spike-arcs for RBP-complex without motif are in orange, red, and brown color. The complexes with the prime motifs were found to be much more stable than their counterparts without the prime motif.

Depending upon the availability of complete experimentally validated 3D structures in PDB database (Rose et al., 2011), structures for 13 RBPs (IGF2BP1, DIS3L2, CNBP, SRSF3, FKBP4, KHDRBS1, LIN28A, CAPRIN2, DICER1, GTF2F1, HNRNPC, CPSF6, and AGO2) were selected for the structural interaction analysis for the identified binding sites. Comparative analysis of RMSD trajectories of 13 different RBPs-RNA complexes with three replicates each for the two conditions clearly suggested that the presence of the identified prime motifs was giving stability to the RBP-RNA complexes (Figure 9).

For example, in the case of AGO2, on the comparative analysis of RMSD value for the AGO2-RNA first sequence complex with the prime motif, the value ranged from 0.1 nm to 0.7 nm and stabilized at 0.5 nm. The RMSD value for the complex without the motif ranged from 0.1 nm to 1.7 nm and stabilized at 1.5 nm, which was less stable. Similarly, the second pair with the prime motif had RMSD values ranging from 0.1 nm to 1.7 nm and it stabilized at 0.6 nm, whereas the same pair without the prime motif had RMSD ranging from 0.3 nm to 1.4 nm and stabilized at 1.4 nm. For the third pair, the AGO2-RNA complex with the prime motif showed deviation from 0.1 nm to 1.0 nm and settled at 0.7 nm, whereas the same sequence without the prime motif showed deviation from 0.0 nm to 2.0 nm and settled at 1.4 nm. In all the three cases of AGO-RNA complexes, the sequence with the prime motif was found to be more stable when compared to the one without the motif in the dynamic environment. A similar pattern was observed for all the 13 RBP and their triplicate pairs. The associated details are given in Table 1.

**Table 1:** Table for RMSD values for the selected 13 RBPs complexes, with and without the prime motif

RBPs name	Sequence name	RMS deviation range value with motif (nm)	Stablized_RMSD value with motif (nm)	RMS deviation range value without motif (nm)	Stabilized RMSD value without motif (nm)
AGO2	RNA_seq1	0.1–0.7	0.5	0.1–1.7	1.5
	RNA_seq2	0.1–1.7	0.6	0.3–1.4	1.4
	RNA_seq3	0.1–1.0	0.7	0.1–2.0	1.4
CAPRIN2	RNA_seq1	0.1–0.3	0.2	0.6–2.1	1.9
	RNA_seq2	0.3–1.8	1.3	0.7–2.2	1.5
	RNA_seq3	0.2–2.1	1.8	0.1–2.9	2.7
CNBP	RNA_seq1	0.1–1.3	1.1	0.3–4.8	4.3
	RNA_seq2	0.3–1.8	0.5	0.3–2.5	2.0
	RNA_seq3	0.1–2.7	2.5	0.1–4.7	3.4
CPSF6	RNA_seq1	0.3–1.2	1.0	0.3–3.2	3.0
	RNA_seq2	0.3–2.0	1.8	0.3–2.8	2.4
	RNA_seq3	0.1–2.9	2.7	0.1–4.8	4.1
DICER1	RNA_seq1	0.3–1.3	1.0	0.3–3.5	2.9
	RNA_seq2	0.3–1.5	1.5	0.3–2.7	2.0
	RNA_seq3	0.1–2.5	2.3	0.1–4.8	4.2
DIS3L2	RNA_seq1	0.1–0.3	0.3	0.3–2.5	2.5
	RNA_seq2	0.3–1.8	1.2	0.3–2.4	2.0
	RNA_seq3	0.1–2.3	1.8	0.1–4.2	4.0
FKBP4	RNA_seq1	0.3–1.5	1.3	0.3–1.2	1.3
	RNA_seq2	0.1–1.7	1.1	0.1–2.5	2.2
	RNA_seq3	0.1–1.9	0.5	0.1–2.3	2.2
GTF2F1	RNA_seq1	0.3–1.3	1.3	0.3–3.7	2.5
	RNA_seq2	0.3–1.9	1.7	0.5–2.5	2.0
	RNA_seq3	0.1–2.6	2.1	0.1–4.7	4.0
HNRNPC	RNA_seq1	0.1–0.5	0.3	0.1–2.7	2.3
	RNA_seq2	0.1–2.3	1.8	0.1–2.3	2.0
	RNA_seq3	0.1–2.5	2.2	0.3–4.3	4.1
IGF2BP1	RNA_seq1	0.3–1.7	0.9	0.3–2.6	2.1
	RNA_seq2	0.3–1.5	0.6	0.07–1.7	1.3
	RNA_seq3	0.1–1.7	1.5	0.1–2.6	1.6
KHDRBS1	RNA_seq1	0.3–1.9	0.5	1.0–4.8	3.5
	RNA_seq2	0.3–1.2	1.2	0.3–2.1	1.2
	RNA_seq3	0.3–2.5	2.5	0.1–4.7	4.2
LIN28A	RNA_seq1	0.3–1.4	0.7	0.3–3.0	2.2
	RNA_seq2	0.5–2.1	1.5	0.5–3.0	1.6
	RNA_seq3	0.1–2.2	2.2	0.1–4.9	3.1
SRSF3	RNA_seq1	0.2–0.5	0.8	0.5–3.7	3.0
	RNA_seq2	0.3–1.2	1.2	0.5–2.5	2.1
	RNA_seq3	0.1–3.0	2.5	1.0–4.8	3.2

The identified prime motifs were found statistically enriched in the target regions when compared to the random regions. Molecular dynamics studies with and without these motifs clearly suggested their important role in binding where they were found responsible for stable complex formation between RBP and RNA.

In the nutshell, the structural molecular dynamics study supported the identified binding spots for the RBPs where it was clearly evident that the identified binding motif provided structural stability to the considered RBP-RNA complexes.

### Application: SARS-Cov2 genome was found to host RBP binding sites

Most of the deadly viruses are RNA viruses which exploit the host proteins to replicate, spread, and survive. The best living example is nSARS-CoV-2. The emergence of the novel human coronavirus SARS-CoV-2 in Wuhan, China, has caused a pandemic of respiratory disease (Covid-19). The big scientific concern is that to this date very scarce and uncertain molecular information is available about the Covid-19 patient’s molecular system as not many high-throughput studies have been carried out so far. There is almost absolutely no information on the host RBPs’ response during Covid-19 infection despite the fact that all such viruses essentially require host RBPs to survive and replicate. The RBP-RNA interaction studies hold prime importance in this regard also.

**Figure 10:**
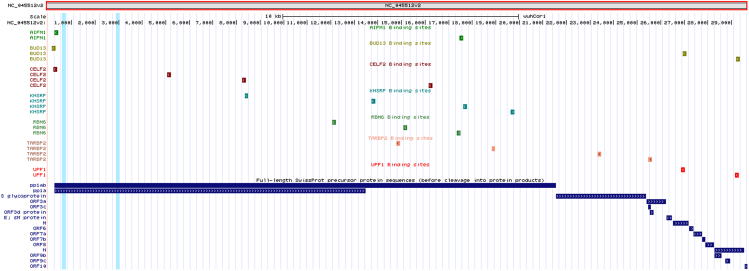
Application of RBPSpot reports the binding sites for seven different RBPs across the nSARS-CoV2 genome A total of 22 such binding sites were discovered across the nSARS-CoV2 genome, all of which existed across the negative strand of the virus genome.

Therefore, we scanned the SARS-CoV-2 genome through RBPSpot to find the binding sites for RBPs which could have some therapeutic value. Interestingly, out of 131 RBPs we found 22 different binding sites for seven different RBPs (AIFM1 (2), BUD13 (3), CELF2 (4), RBM6 (3), UPF1 (2), TARBP2 (4) and KHSRP (4)) (Figure 10). Among these, AIFM1’s interaction with the viral polymerases in influenza virus infected cells is well studied (Bradel-Tretheway et al., 2011). All these binding sites were found across the anti-sense strand of the genome whose importance is for the viral replication. During the infection, the majority of immunoprecipitated RNAs of the corona virus were found originating from the anti-sense strand (Hackbart et al., 2020). Thus, there might be a possibility that these RBPs are helping in the viral transcription by binding to its negative strand. To check the stability of these RBPs with their binding site, an MD simulations study was performed on the two different sequence forms for each identified binding site (one with the binding site motif and another one without it). Prior to this, the complete 3D structures for AIFM1 and UPF1 were obtained from PDB while the remaining five RBPs were modeled through homology modeling due to lack of complete defined structures for them. After the modeling, the built 3D structure models were evaluated using SAVES v6.0 (structure Activity validation server). Five RBPs PDB structures viz. AIFM1, BUD13, CELF2, TARBP2, and UPF1 passed through the verification filter such as PROCHECK and WHATCHECK except KHSRP and RBM6. When the model structures for KHSRP and RBM6 were analyzed with both the programs, 80.9% and 83.5% of the residues were found in the allowed regions in the Ramachandran plot, respectively. However, for a good quality model over 90% residues are expected in the most favored region and with the lack of loop filtering causing side-chain packing inaccuracies. Subsequently, upon analyzing the RMSD graph (Figure S3) for all the seven RBPs, it was found that five out of seven RBP-RNA complexes were stable with their respective prime motif when compared to the RBP-RNA complexes counterpart without the prime motif. This part of the study was done just to showcase the application of the developed approach. The finding made in this section may be used for further study by the Covid research groups.

**Figure 11:**
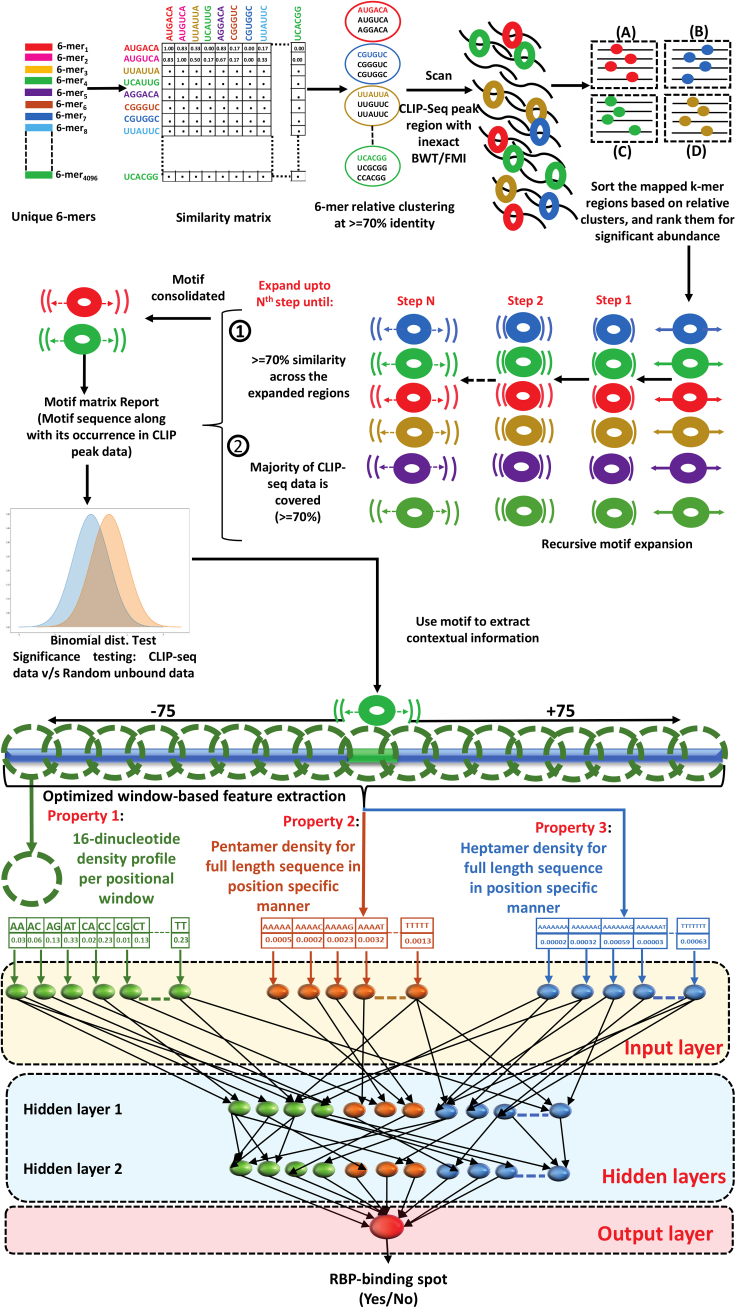
Illustration of the basic workflow of RBPSpot This illustrates the brief outline of the entire computational protocol implemented to develop the accurate RBP RNA interaction models and identify the correct RBP binding sites across given RNA sequences. The process of model building starts from identifying the prime motifs through k-mer spectrum search from the CLIP-seq regions where BWT/FM indexing based inexact search algorithm was implemented. The statistically enriched k-mers were expanded across all reporting sequences regions until there was at least 70% similarity between them. The final prime motifs were established as the anchors. The flanking regions around such anchored prime motifs were used to derive the contextual information together, which worked as feature vector elements for discrimination using XGBoost and Deep Learning.

With the advent of high-throughput techniques like CLIP-seq and interactome capture, the information on genes recognized as RBPs and their interactions are growing continuously. Such high-throughput data on interactions are very valuable resources to construct the interaction models. The present study used the same from CLIP-seq experiments. However, there are several other critical factors involved which are required to build these interaction models with high accuracy. This involves proper negative datasets screening, appropriate motif discovery strategy which could anchor for correct context, and the appropriate contextual information itself. All of them are interconnected with each other and the success of any such RBP binding site discovery tool depends highly on this. When all this information is applied through some effective machine learning algorithms, a consistently high level of accuracy is achievable. Figure 11 illustrates the overall implementation of the developed algorithm and software in the present study. The developed software was comprehensively and comparatively benchmarked against some recent tools where it outperformed the compared tools consistently across a wide number of datasets and RBPs. It also showcased that when a DNN is trained properly on suitable properties with appropriate biological insights, the developed system could easily outperform much complex deep-learning based approaches where such learning is done through an automated feature extraction process using complex layers like CNN and LSTM. Such complex deep-learning approaches may be suitable for unstructured data where features cannot be identified easily. However, when features are identifiable and structured, simpler machine learning approaches can outperform them easily. The developed approach in this study, RBPSpot, can identify the binding sites of existing RBPs in the human system. Also, it becomes one of the few tools where the users may input their own data and raise their own models for any species and any RBP. The software is freely available as a web server and as a standalone program.

### Limitations of the study

RBP RNA interaction is also very spatiotemporal in nature and controlled in a networked fashion. At present, the existing tools including the present one do not consider the spatiotemporal factors to precisely measure the degree of interaction and regulation for any given condition. From here, we visualize the incorporation of spatiotemporal and other interactome network information for RBPs as another dimension to explore to further improve our understanding on RBP-RNA interactions. This is something which still remains largely unaddressed. There have been some encouraging recent developments (Pradhan et al., 2021; Mukherjee et al., 2019) that suggest that incorporation of back-end networks and interaction information on RBP RNA interactions may help develop the models which could also involve functional and dynamic nature of RBP RNA interactions and predict the spatiotemporal behavior of the interactions. Also, the findings made here from contextual information such as CG enrichment in the flanking regions may be explored further for their functional roles associated with such binding sites. RNA modifications on CG and, likewise, other important contextually important factors found in this study may further provide more reasoning for the spatiotemporal nature of these interactions. That would mark another level of development in our understanding toward RBP-RNA interactions and regulation. Recently, a multiple kernels based CNN was applied to use the associative information of motifs for the classification of nucleic acid binding proteins (Zhang et al., 2020). In a similar manner, the significant motifs found in the present study besides the prime motif may be used in a combined manner to build multiple motifs’ PWM based convolution kernels in the parallel to derive and utilize their mutual association information specifically. Although the complexity may increase as already discussed above with the complex deep-learning approaches, such a system may be integrated in parallel to the presented RBPSpot approach to build a hybrid system to further bolster the contextual information derived by RBPSpot. Also, many new machine learning approaches now arrive frequently which may be routinely explored for the release of the upgraded versions of RBPSpot in the future.

## STAR★Methods

### Key resources table

**Table:** 

REAGENT or RESOURCE	SOURCE	IDENTIFIER
**Deposited data**		

Code availability	Github	https://github.com/SCBB-LAB/RBPSpot

**Software and algorithms**		

CLIP-seq peak data	Li et al., 2014	http://starbase.sysu.edu.cn/ and Table S13
Python3	Python	https://www.python.org/
Numpy	Numpy	https://numpy.org/
Pandas	Pandas	https://pandas.pydata.org/
perl	perl	https://www.perl.org/
sklearn	sklearn	https://scikit-learn.org/stable/
XGBoost	XGBoost	https://xgboost.readthedocs.io/en/latest/
bedtools	Quinlan lab	https://bedtools.readthedocs.io/en/latest/
keras	keras	https://keras.io/
tensorflow	tensorflow	https://www.tensorlow.org
gcc	gcc	https://gcc.gnu.org/
Human genome file	UCSC	https://genome.ucsc.edu/
SARS-Cov2	NCBI	NC_045512

### Resource availability

#### Lead contact

Further information on resources and reagents should be directed to the lead contact, Ravi Shankar (ravish@ihbt.res.in).

#### Materials availability

This study did not generate new unique reagents.

### Experimental model and subject details

No experimental model system used for this study.

### Method details

#### Data retrieval and processing

CLIP-seq peak data from various sources were collected for 137 RBPs using starBase 2.0, also known as ENCORI (Table S13). All the data were collected in the form of coordinates. These data were from multiple types of CLIP-seq experimental techniques, i.e., CLASH, dCLIP, eCLIP, FLASH-CLIP-seq, HITS-CLIP, iCLIP, PAR-CLIP, sCLIP-seq, and uvCLAP. The peak data varied from 234 (PAPD5) to 984,503 (U2AF2) peaks. Initially, six RBPs’ data were discarded due to insufficient peak data availability. Here, we considered only those RBPs which were having >500 unique binding peaks available. These six RBPs *viz.* PAPD5 (234), EIF3B (298), EIF3A (371), EIF3G (398), EIF3D (399), and PUM1 (473) had fewer number of initial peaks available. Remaining data for 131 RBPs were having a total number of 21,123,594 unique peaks. To further filter this data, we discarded those sequences which were having a length <5 bases or extreme length sequences (>300 nucleotides). With this all, a total of 18,714,999 peaks were available for the study, varying from EIF4A1 (1,175) to AGO1-4 (9,41,224). Initial coordinate data were extracted into sequences from genome. Initial and final data are given in (Table S14).

#### Identification of motif seed candidates: A modified BWT/FM-Indexing based inexact search

The CLIP-seq peak regions were scanned to find out the most abundant 6-mers seeds with two mismatches allowed in the initial step of motif finding. The reason behind this was that some of the previously reported motifs for RBPs were either very sparse or as small as four bases long. Therefore, a k-mer with six bases and with two mismatches would fetch all the possible 6-mers spectrum which would agree with each other with two mismatches (relatives to the main k-mer) while also meeting the lowest bound of such motifs. This defined the 6-mer groups within two mismatches. Iteratively, and in parallel, the generated k-mer spectrum for each such sequence was searched across all the reported cross-linked associated regions in the targets to obtain the enrichment status of the k-mers (seeds) on which the motif would be built. These searches were allowed with a maximum of 30% mismatches. Since a large number of 6-mers spectrum is created whose search with two mismatches across the sequences becomes a computationally intensive and time-consuming step, an FM-Indexing and Burrows-Wheeler Transformation (BWT) based inexact search step was applied. Since the parallelism through multiprocessing was also implemented, the search linearly increased with the available CPU cores. The detailed implementation pseudo-code of the Burrows-Wheeler Transformation and FM-index based search algorithm is given below:

A sequence ‘S of length len(s) is made of a set of alphabet letters *Φ* = {A, C, G, U}. S*[i]* denotes the *i*th position of base as (0<=i<=len(s)-1) and S*[i,j]* represents a sub-sequence which starts at position ‘*i*’ and ends at ‘*j*’. Thus, each such sequence region of length len(S) generates len(S)-6 k-mer sub-sequences of length of six bases which is converted into a set of unique 6-mers. Initially, all of them are assumed to be a potential candidate, which could give birth to a motif seed which becomes clear after a continuous search across all the CLIP-seq peak regions for every such candidate and enrichment analysis for over-representation. Mapping each such sub-sequence query across the reference sequence *‘*S*’* (CLIP-seq peak regions) required two basic operations: 1) Finding the frequency of the query sub-sequence ‘*q’* in the reference sequences, and 2) locating the position of the sub-sequence ‘*q’* in the reference sequence ‘S’.

Step 1: Input: k-mers set as set of queries ‘q’ and Set of reference sequences ‘S_N_’
Step 2. Perform BWT transformation of ‘S_N_’_._
Step 3. Make the dictionary of count of all the bases by using BWT and use it to find the tally dictionary.
Step 4. Make the tally matrix using FM index based search of k-mer.
Step 5. Using BWT transformed ‘S_N_’ set of reference sequences, generate the base count dictionary and tally dictionary. The tally dictionary stores the base position records.
Step 6: Repeat:
Step 7: Keep updating the tally dictionary according to the found base positions.
Step 8: Until the cycle reaches the end reference sequence, for all elements of ‘S_N_’.
Step 9: Generate the final first column dictionary holding base and position in sorted manner.
Step 10: Search query k-mer ‘*q’* in the reference set using first column dictionary and tally dictionary.
Step 11: Return: Report all found locations.

The inexact search is done further by generating all the possible k-mers with given mismatch level to the root k-mer. These similar relatives are parallelly searched across the generated indices through the above-mentioned steps.

#### Identification of motif seeds candidates: Anchoring with the significant seeds

All the k-mer seeds and their relatives displaying at least 70% similarity were evaluated for their existence across at least 70% of peak data. Such motif seed candidates were further evaluated for their statistical significance. Those RBPs where no k-mer and their relatives crossed 70% representation in the data were looked for the highest representation available. The remaining data which did not show the representative k-mer were checked further and recursively with minimum cut-off of 20% data representation. Motifs coming from such data were considered as mutually exclusive ones. Null model distribution probabilities of occurrence of each k-mer along with its relatives were calculated from the random data set to find their random probabilities. Random data set was generated from unassociated RNAs while randomly carving out the lengths similar to the peak data. Significantly over-represented k-mers were screened using binomial test with p-value cut-off of 0.01. These significantly enriched k-mers were used as initial seeds to develop the final motif. These seeds of significantly enriched k-mers were expanded in both the directions by expanding by one nucleotide both sides, followed by search across the peak data with at least 70% occurrence in the peak data while repeating the above-mentioned search operation recursively. Expansion of seed region in both directions was allowed until at least 70% match existed. The final motifs were selected on the basis of satisfying both the criteria, i.e. the motif displayed at least 70% abundance across the CLIP-seq instances at 1% significance level, and the maximum k-mer expansion maintained at least 70% identity with the associated sequences and relatives. Mutually exclusive motifs were other predominant motifs which existed in the remaining data which were scanned in a similar recursive manner as described above (Tables S1 and S2).

#### Datasets creation

Once we had the prime motifs anchored for each RBP from the given data, their associated peak data sequences were converted into the positive datasets. Thus, uniform length sequences with flanking regions were obtained for every individual RBP which varied for different RBPs depending on the length of their anchor motifs. To generate the positive datasets for each RBP, start and end coordinates from the prime motif’s two terminals were expanded by +75 and −75 bases into both directions. In the case of multiple motif locations originating from a single peak for the main motif, all the locations were expanded. Different length dataset sequences formed for different RBPs which depended mainly upon the length of the core motif region. However, for any single RBP all the sequences of the dataset were of the same length. This also led to the construction of positive and negative instances datasets covering a total of 19,547 genes experimentally confirmed as targets of these RBPs. The total number of instances was greater than the total number of peak data for most of the RBPs due to the multiple occurrences of motifs on a single sequence.

Identifying suitable negative dataset candidates becomes a more crucial task, and it is where most of the previously developed tools have gone too soft and mostly ended up selecting random sequences, which actually does not help to divulge more information. As transpires from the discussions and results section on motifs, there are many spots across the transcriptomes which possess sequence similar to the interaction motifs, but they yet not interact. Usually, chances of finding shorter motifs themselves are higher in the random data. In such scenario, considering random sequences really does not add significantly to the purpose of discrimination and does not answer the question raised above. In order to build a better negative dataset, it is better to pick those candidates as negative instances where the region similar to the main motif is present. This creates strong confusion matrices to build a more natural and robust model. Therefore, to create the negative set for RBPs two different kinds of strategies were used.

In the first strategy we used RNA-seq data for the same condition for which we had the cross-linking data available for the given RBP. Those RNAs were selected which were expressing themselves in the same condition but did not bind to the considered RBP and did not reflect in the CLIP-seq data. Also, it was ensured that the considered transcripts had minimum three replicates of RNA-seq data for which expression was calculated. This data was collected from GEO. They were searched for the prime motifs of the RBP similar to the positive data cases and, in a similar manner, 75 bases flanks were considered along with capturing the contextual information with more discrimination power. For the RBPs for which the negative datasets were created using this strategy were called Set A RBPs datasets throughout this study. This way, the negative datasets for 74 RBPs were created (Table S5). Strong negative datasets were built which ensured that learning was in no way influenced by the motif alone as the motif may also occur randomly to some extent, and the surrounding context is also considered along in a right manner.

In the second strategy, negative datasets were created for those RBPs which did not have similar condition RNA-seq data available for the considered CLIP-seq conditions. Therefore, in such scenario those RNAs were considered which exhibited binding to their respective RBPs but also had the motifs on other positions which did reflect in the CLIP-seq data and were also far away from such cross-linking regions. The logic behind this is that such RNA sequences in which some regions exhibited binding to RBPs in CLIP-seq data make clear positive instances out of these regions as well as hold simultaneous evidence that these RNAs were expressed in the given condition. Regions which display the prime motif in these expressed RNAs but do not bind to the RBPs become an apt case for negative instance consideration with high potential for contextual information unlike the usual random sequences. For 57 RBPs similar RNA-seq data were not available for the corresponding conditions. In such a scenario the main motifs for negative datasets were searched in those regions which did not appear in the CLIP-seq data but belonged to the same target RNA sequences in which some parts appeared in the CLIP-seq. In this manner, although the RNA was expressed and even bound to the RBP, these regions despite having the motif for the RBP did not bind to the RBP and were suitable for negative dataset formation. +75 and −75 flanking bases from both the terminals of the motifs were considered along with the motif region to build the negative datasets. These datasets were called Set B datasets. In this way, the Set B negative dataset were created for the remaining 57 RBPs (Table S6).

#### Feature generation for positive and negative datasets

Five different types of properties were considered for input into machine learning: 1) The main motif itself, 2) Dinucleotides density in the associated region while considering 75 bases flanking regions from both the sides of the motif, 3) Dot-bracket representation of the RNA structural triplet for the dataset sequences, covering 27 combinations of structure triplets arising from the dot-bracket structural representation from RNAfold predicted RNA structures [.((, .(), .(., .)(, .)), .).,..(, ..), ..., (((, ((),((.,()(,()), ()., (.(,(.), (.., )((, )(), )(.,))(, ))), ))., ).(, ).), ).., ] , 4) Pentamers density profile for each position which captures the shape information, and 5) Heptamers densities for the complete region. The dinucleotide densities were evaluated for their discriminatory power for multiple sliding windows starting from 17 to 131. Similarly, the dot bracket structural triplet representations of the dataset sequences were generated using RNAfold (Lorenz et al., 2011). They too were evaluated for the optimum windows size while testing for the window sizes ranging from 29 to full sequence for the given window size. A total of 1,024 pentamers and 16,384 heptamers densities were evaluated in a similar manner across the dataset sequences.

To calculate the heptamers based feature, all the positive datasets were split into k-mers of seven bases. The probability of each k-mer was calculated with a maximum of two mismatches for each position and accordingly populated in the tensor. Thus, we had 16,384 X ((sequence length) - 7) tensor of probabilities. 16,384 rows represented the heptamers and 150 columns represented the individual positions. In a similar manner, pentamer features were calculated. For that we had 1,024 X((sequence length)-5) tensor of probabilities. These both tensors were used to convert the sequence data into vectors of probabilities. Altogether, based on the optimum windows the combined features sets representation of all the dataset sequences was achieved. The optimum windows and total features varied for each RBP. Finally, each dataset was broken into training and testing datasets ensuring that no instances from training ever appeared in the testing datasets. The breakup for each RBP for their training and testing datasets is given in Tables S5 and S6.

Consideration of heptamers was for picking up any further sequence specific signals in the flanking regions. An approach similar to the above-mentioned inexact search was applied with at least 70% similarity match as was done for the prime motifs' 6-mer seeds. The pentamers application was motivated from the recent findings which reported that the pentamers capture the DNA shape very accurately (Zhou et al., 2013). The nucleic acids shape has been found critical in the interactions with regulatory proteins which scan these shapes for their stationing. So far, this approach has been applied on DNA but hardly on RNAs. The DeepBind work had observed about the importance of using such kind of information which could be beneficial in future developments for the tools reporting RBP-RNA interactions (Alipanahi et al., 2015). The dinucleotide densities have been found to be highly useful in indirectly evaluating the RNA structure and accessibility (Heikham and Shankar 2010; Černý et al., 2020). In fact, it has been found more promising than *ab-initio* RNA structure prediction. *Ab-initio* methods’ accuracy drastically falls with the length of RNA, and they are suitable for only short RNA sequences (Paz et al., 2014; Heikham and Shankar 2010). Pentamer and dinucleotide frequencies capture better structural and shape information through base stacking and neighborhood contribution. Similarly, RNA structure triplet has been used widely in deriving the structural information of RNA for their propensity toward interaction factors, especially for miRNA:RNA interactions (Xue et al., 2005).

#### Features evaluation on datasets

After generating all the features from positive and negative datasets, they were individually checked for their performance using tree-based approaches which are expected to perform better on high dimension instances. Random forest and XGBoost were applied. Each property and their associated feature sets were evaluated for the varying window sizes for their discrimination power between the positive and negative sets. Sliding windows of variable sizes were used for dinucleotide and structure-based features. These variable sized windows were evaluated for the performance. Out of these different sized windows, the size producing the best performance was kept for final model generation. It was found that the best performing window size varied across the RBPs, resulting into different optimum windows for the RBPs.

Pentamers and heptamers densities appeared as the most informative on the full length window. Equal numbers of positive and negative instances were chosen for all RBPs considered in the study. From the total chosen instances, 60% were used to create the training set, while remaining 40% instances were used to create the testing set. Python scikit-learn library was used for the same purpose. For feature importance evaluation F-score was used for every considered feature. F-score locates the features which display major difference between their values between negative and positive training sets while comparing the averages for the feature values for positive, negative, and whole set of instances (Chen and Lin 2006). The F-score is represented by the following equation:

$$F\left(i\right)=\frac{{\left({\left(\bar{x_{i}}\right)}^{+}-\bar{x_{i}}\right)}^{2}+{\left({\left(\bar{x_{i}}\right)}^{-}-\bar{x_{i}}\right)}^{2}}{1/\left(n_{+}-1\right)\sum_{k=1}^{n^{+}}{\left(x_{\left(k,i\right)}^{+}-\bar{{\left(x_{i}\right)}^{+}}\right)}^{2}+1/\left(n_{-}-1\right)\sum_{k=1}^{n^{-}}{\left(x_{\left(k,i\right)}^{-}-\bar{{\left(x_{i}\right)}^{-}}\right)}^{2}}$$

where F(*i*) = Feature score for the ith feature, ${\left(\bar{x_{i}}\right)}^{+}$ = Avergae for *i*th feature across the positive instances, $\bar{x_{i}}$ = Total average of the *i*th feature across the complete dataset, ${\left(\bar{x_{i}}\right)}^{-}$ = Average for *i*th feature across the negative instances, $x_{\left(k,i\right)}^{+}$ = Feature value for *k*th instance for *i*th feature in positive dataset, $x_{\left(k,i\right)}^{-}$ = Feature value for *k*th instance for *i*th feature in negative dataset, *n*_*+*=_ Total number of positive instances, *n*_−_ = Total number of negative instances.

Also, for every *i*th feature, t test was conducted between *n*_*+*_ *and n*_−_ to evaluate the significance of *i*th feature for its discrimination capability between positive and negative instances.

#### Machine learning implementation

With the optimized windows in the above mentioned section, feature vectors for all the RBPs were used to build models to recognize RBP binding sites using two major machine-learning approaches: XGBoost and Two Hidden Layers based Deep Feed Forward Neural Networks (DNNs). Both were implemented using python scikit-learn, Keras, and Tensorflow libraries. In both the cases 70% and 30% of data were retained for train and test sets, respectively.

The DNNs were built where the input layers had the number of nodes equal to the number of features for the RBP considered. Thus, the size of input layers varied from 1,200 to 2,500. The performance of DNN was also evaluated for various numbers of hidden layers where finally total two hidden layers were found performing the best. The connections between the nodes were made dense. For every RBP model the number of nodes across the two hidden layers varied between 700 and 1,300. Different types of activation function combinations were applied for the layers from a pool of a number of available activation functions. The activation functions define the layers and transform the activation values obtained from previous layers to a non-linear form, creating several hyperplanes to obtain the best possible discrimination of instances. In most of the models here, the first hidden layer had RELU and the second hidden layer had ELU (for some cases they interchanged also), while the final output layer had sigmoid function.

Every learning step provided an estimation of the error made, measuring the error and accordingly correcting the learning rate and weights on connections. This error estimation was achieved by loss/cost functions. Multiple types of loss functions were tried to optimize the accuracy. The best performance was obtained for Binary Cross Entropy. Since it is a feed forward network where the cost function assess the missed targets and accordingly network connection weights are updated though some optimizer. The optimizer parameter which worked the best was ‘Adam’ optimizer, otherwise SGD with momentum. Usually Adam optimizer works better because of its capability to provide different learning rates per parameter, deals better with sparse gradients, and adapts based on recent learning rates while keeping them in memory. Momentum was applied in the learning which helps to ward-off entrapment under local minima during the minimization steps. The learning rate varied from 0.001 to 0.01 and momentum varied from 0.05 to 0.9. L1 and L2 weight decay regularizers were applied to avoid over-fitting. DNN models were trained using 1,000 epochs and batch sizes varied from 50 to 200 instances. All the models from DNN and XGBoost were saved in protobuf format. Since the entire system was implemented here using TensorFlow, the protbuf file provided the graph definition and weights of the model to the TensorFlow structure. The optimum parameter values were fixed using an in-house developed script which tested various combinations of the values of the parameters to pick the best ones. The complete list of the optimized hyperparameters for all the 131 RBP models is given in the Table S15.

In XGBoost, a grid search was applied for parameter optimization. Following parameters were finalized after the grid search: params = {"eta/learning rate": 0.2, "max_depth": 4, "objective": "binary:logistic","silent": 1, "base_score": np.mean(yt), 'n_estimators': 1000, "eval_metric": "logloss"}. Gradient boosted decision trees learn very quickly and may overfit. To overcome this shrinkage was used which slows down the learning rate of gradient boosting models. Size of the decision tree was run on max-depth= “9”. At the value of “4” stability was gained as the log-loss value stabilized and did not change, thereafter.

To evaluate the consistency of the performance of the models developed with the given features, 10-fold cross validation was also performed for each RBP. Every time the training dataset was split into 70:30 ratio with the first part used to train and the second part was used to test, respectively. Each time data was shuffled and random data was selected for building new model from scratch. This process was repeated 10 times for each RBP. Accuracy and other performance measure were calculated for each model (Table S16). In order to avoid any sort of imbalance, memory, and bias, it was ensured that no similarity existed ever between the train and test sets for any performance measure done here. Also, it was ensured that every dataset in the study carried an equal number of representations for both positive and negative instances.

The performance on test sets was also evaluated. Confusion matrices containing correctly and incorrectly identified test set instances were built for each RBP. Frequently used measures for classifier performance evaluation and accuracy of RBPs models were evaluated. Sensitivity (Sn)/Recall/True Positive Rate (TPR) defines the portion of positives which were correctly identified as positives, whereas the specificity describes the portion of negative instances correctly identified. Precision estimates the proportion of positives with respect to total true and false positives. F1-score was also evaluated which measures the balance between precision and recall. AUC/ROC was also measured for each model. Besides these metrics, Matthew’s Correlation Coefficient (MCC) was also considered. MCC is considered among the best metrics to fathom the performance where score equally influenced by all the four confusion matrix classes (true positives, false negatives, true negatives, and false positives) (Chicco and Jurman 2020). A good MCC score is an indicator of robust and balanced model with high degree of performance consistency.

Performance measures were done using the following equations:

$$\text{Acc}=\frac{\text{TN}+\text{TP}}{\left(\text{TN}+\text{TP}+\text{FN}+\text{FP}\right)}$$

$$\text{Specificity}\left(\text{Sp}\right)=\frac{\text{TN}}{\left(\text{TN}+\text{FP}\right)}$$

$$\text{Precision}=\frac{\text{TP}}{\left(\text{TP}+\text{FP}\right)}$$

$$\text{Recall}/\text{Sensitivity}\left(\text{Sn}\right)=\frac{\text{TP}}{\left(\text{TP}+\text{FN}\right)}$$

$$F1-Score=2\times (\frac{Precision\times Recall}{Precision+Recall})$$

$$\text{AUC}=\int_{\text{0}}^{1}\text{Pr}\left[\text{TP}\right]\left(v\right)\text{dv}$$

$$\text{MCC}=\frac{\text{ TP}\times \text{TN}-\text{FP}\times \text{FN}}{\sqrt{\left(\text{TP}+\text{FP}\right)\left(\text{TP}+\text{FN}\right)\left(\text{TN}+\text{FP}\right)\left(\text{TN}+\text{FN}\right)}}$$

where TP = True Positives, TN = True Negatives, FP = False Positives, FN = False Negatives, Acc = Accuracy, AUC = Area Under Curve.

#### Structural analysis and MD simulations of the identified binding spots

To assess the stability and dynamics of the RBP-RNA complexes for the identified binding spots, a structural analysis was done. The 3D coordinates of RBPs were retrieved from the Protein Data Bank (PDB). X-Ray crystallographic structures for 13 different RBPs were downloaded. Prior to docking, protein structures were prepared by removing the water molecules and other hetero-atoms while adding polar hydrogen atoms. The prime binding motifs identified through RBPSpot algorithm for above-mentioned five RBPs were taken as flexible molecules. All docking studies were performed through NPDock (Nucleic Acid–Protein Docking) and PATCHDOCK incorporating more realistic DARS-RNP statistical potential based on reverse Boltzmann statistics to score protein-RNA complexes (Tuszynska and Bujnicki 2011). RNA motifs three dimensional structures were built using RNACOMPOSER web server based on RNA FRABASE database relating the RNA secondary and tertiary structure elements. In order to search for all possible RNA-binding sites and optimize the structural effects of RNA on the construction of complex, short RNA motifs were taken into account. Protein-RNA interface residues were predicted using DR_Bind1 (Chen et al., 2012) based on evolutionary conservation. Top three representative docking potential-ranked protein-RNA complexes were built for each of the above-mentioned RBPs and the best one was considered for further analysis.

In order to examine conformational variations of the RBPs within the hydrated controlled environment, the root-mean-square deviation (RMSD) of the atomic positions of RNAs containing motif with respect to RBP backbone were calculated and compared with the RNA complexes without the prime motif. In the comparative analysis of RMSD measures these RBPs complexes were considered with three different RNA sequences for each RBP. These sequences were randomly selected from positive datasets having 75 bases flanking regions around the prime motif region. To analyze the structural behavior of RBPs and their complexes, 20 ns simulation job was performed. For this purpose, selected RBPs and complexes were immersed in the cubic boxes of varying dimensions based on the system size. Prior to the energy minimization process, different charged molecules like NA^+^ or Cl^−^ were added to neutralize the system (Pfeiffer et al., 1999).

All molecular dynamics simulations of the RBPs alone and the RBP–RNA complex were conducted using GROMACS 5.1 package (Berendsen et al., 1995), modeling each system with the AMBER03 force-field of protein and nucleic acids (Duan et al., 2003) with periodic boundary conditions. RMS module in GROMACS was executed while choosing “Backbone” for least-squares fitting and “RNA_Heavy” for the RMSD calculation. By doing so, the overall rotation and translation of the protein was removed via fitting. The RMSD reported about how much the RNA position varied relative to the protein. This is considered as a good indicator of how well the binding pose was preserved during the simulation. The topology files for the selected target RNA motifs were built using pdb2gmx in the framework of AMBER03 force-field. Models were solvated with the TIP3P water model (Jorgensen et al., 1983). The distance between the biomolecule and the edge of the simulation box was set as minimum 1.0 Å so that they could not directly interact with their own periodic boundary condition and fully immerse with water while rotating freely. Boxes were solvated with TIP3P water. The number of solvated molecules added to each system varied. After the establishment of initial configuration, the systems were minimized. 50,000 steps (steepest descent approach) were used for each system until the maximum force of <10.0 kJ/mol for energy minimization. For the calculation of the long range electrostatic interactions, Particle Mesh Ewald (PME) method was used. To establish the systems at constant temperature of 300K, V-rescale thermostat (modified Berendsen thermostat), at a constant pressure of 1 bar, and Parrinello-Rahman barostat were applied with a 2 ps coupling constant for both parameters. The LINCS algorithm (Hess et al., 1997) was used to constrain all bond lengths involving hydrogen. During the production run, a time step of 2 fs was used and conformations were saved every 10 ps for the analysis of molecular dynamics trajectory of total 20 ns for each RBP and their complexes using leap-frog algorithm (Gunsteren and Berendsen 1988) to integrate the equation of motion. MD trajectories were further evaluated for considering root mean square deviation (RMSD). RMSD is suitable to decipher the structural changes in proteins and their complex structures corresponding to initial structure during the course of different time periods of dynamics simulation. RMSD was calculated using the following equation:

$$\text{RMSD}=\sqrt{\frac{1}{N}\times \sum_{\text{i}=1}^{N}{\left(u_{i}-v_{i}\right)}^{2}}$$

where u_i_ = Cartesian coordinates of atom *i* in the initial structure; v_i_ = Cartesian coordinates of atom *i* in the structure during simulation; N = number of atoms; To analyze the structural properties of the individual RBPs and their complexes in the form of root mean square deviation (RMSD), g_rms functions were utilized. Changes in trajectories of molecular dynamics during course of simulation were plotted for evaluation using python plotting library.

#### Co-occurring RNA motifs group clustering

A two-step statistical approach was employed to identify the co-occurring motif pairs. In this approach, the positive set of RBP was scanned for other more frequent occurring k-mers. Top co-occurring motifs were checked for their statistical significance. KS-test was used to find the significance of distance for two motifs. All the distances between the two motifs were calculated from positive and negative datasets. Distribution plot of random data and positive data were further checked using KS-test. Level of significance was considered p < 0.05. They were further checked for frequency ratio (FR). At 5% level of significance, if the hypergeometric test *p value* was less than 0.05, motif pair of enriched and co-occurring motifs was considered significant. Additionally, frequency ratio (FR) as a measure of co-occurrence of motif pairs was also computed to estimate the tendency of motif pairs to co-occur with each other as proposed previously (Vandenbon et al., 2012):

$$FR\left(Motif_{M2/M1}\right)=\frac{X_{M2/M1}/N_{M1}}{Y_{M2/M1}/M_{M1}}$$

X_M2/M1_ = Number of sequences containing motif1, N_M1_ = Number of sequences containing motif2 co-occurring with motif1, Y_M2/M1_ = Number of sequences without motif1, M_M1_ = Number of sequences containing motif2 without motif.

Co-occurring motifs were also evaluated for Jaccard similarity index calculation to ensure that different motif partners are evaluated instead of the same motif. The method utilizes the position weight matrices of co-occurring motifs for alignment considering relative shifts to recognize similarity between two motifs (Vorontsov et al., 2013).

#### Benchmarking and performance evaluation

To evaluate the RBPSpot performance and the importance of datasets constructed in this study, we compared RBPSpot with five different tools: RBPmap, DeepBind, iDeepE, DeepCLIP, and beRBP. Three different datasets were considered separately for the benchmarking process: Datasets used for RBPSpot, beRBP, and GraphProt. Datasets of beRBP and GraphProt are common data source for most of the existing published software built to identify RBP-RNA interactions. As already mentioned above, the RBPSpot datasets were based on the positive datasets from ENCORI (the encyclopedia of RNA Interactomes, previously known as StarBase) and the negative datasets was based on the protocol mentioned above in the previous section. The RBPSpot datasets contained positive and negative sequences for 131 RBPs in which the length of the sequences varied from the minimum of 156 bases to the maximum of 160 bases. The variation in the length of the sequences for different RBPs was due to the varying length of their major motifs. For the benchmarking purpose, those RBPs data were considered from RBPSpot datasets for which at least one tool had model reported for comparison. No such RBP was considered from the RBPSpot datasets for benchmarking for which no other tool had any model reported for comparison. This way a total of 52 RBP data were used from RBPSpot datasets for the comparison purpose.

The beRBP dataset was available for 29 RBPs. This dataset is based on the experimentally validated target sequences (3′-UTRs) for human RBPs (positive datasets) from AURA (Dassi et al., 2014) (v2, 8/5/2015; http://aura.science.unitn.it/), which is a manually curated and comprehensive catalog of human UTRs bound by regulators including RBPs. Negative instances of this dataset has random sequences chosen from the 3′ UTR pool. The beRBP dataset was obtained from the URL http://bioinfo.vanderbilt.edu/beRBP/download/TabS1.7z.

The third dataset considered in this study was built during the work presenting GraphProt software. Since then, this dataset has been used extensively by many published software to this date. This dataset covers CLIP-seq data for 24 RBPs. For each set of CLIP-seq data, they created a set of unbound sites by shuffling the co-ordinates of bound sites within all genes occupied by at least one binding site which worked as the negative instance for negative dataset. This dataset was retrieved from the URL http://www.bioinf.uni-freiburg.de/Software/GraphProt/GraphProt_CLIP_sequences.tar.bz2.

Four of the five compared tools, *viz.*, beRBP, RBPmap, DeepCLIP, and DeepBind provide pre-built models. Only iDeepE does not provide any pre-built model. To overcome this, models were generated using iDeepE methodology for the datasets. To make binary decisions with DeepBind, threshold of 0.7 was applied after performing logistic transformation of the raw DeepBind scores (Yuan et al., 2019).

In the second part of the benchmarking study, impact of the datasets was assessed on the model building quality when models were built using different datasets and various combinations of test and train datasets were analyzed. Besides RBPSpot, only two tool, iDeepE and DeepCLIP, had provision to build models from user provided datasets. The remaining tools have fixed models with which they work and do not provide the provision to build models from user provided data. Therefore, they could not be included in this part of benchmarking. Thus, for this part, the datasets used by RBPSpot (RBPSpot dataset), iDeepE, and DeepCLIP (GraphProt dataset) were used. Four different combinations of train and test datasets (RBPSpot train + RBPSpot test, RBPSpot train + GraphProt test, GraphProt train + RBPSpot test, and GraphProt train + GraphProt test) were used for the benchmarking to evaluate the impact of datasets on the performance of these algorithms.

#### Comparison with experimentally reported motifs

A total of 29 RBPs from RNAcompete study were found overlapping with our set of 131 RBPs. Their IUPAC motifs were downloaded from the RNAcompete web portal. For these 29 RBPs a total of 44 motifs were reported. Out of these 44 motifs, 35 motifs had a length of seven bases, eight motifs had a length of six bases, and one motif had a length of five bases. Four motifs out of 44 were discarded due to more than three variable positions in a length of seven bases. Therefore, in the final analysis a total of 40 motifs representing 26 RBPs were present. These motifs were scanned in the similar manner as was done with the search for motifs identified by RBPSpot approach in order to maintain an unbiased motif search approach. Random data sets to evaluate the random chance observations were generated from the transcriptome data using the length exactly similar to the ones from the cross-linking peak data. The similar above mentioned allowed mismatches based motif searching criteria was used here also to scan the random datasets for motif occurrence in them. Binomial test was applied to find the significance of these motifs in the cross-linking data. Other than RNAcompete motif, experimentally validated motifs were also considered from CISBP-RNA Database. A total of 31 RBPs from this dataset were found overlapping with RBPSpot data. Of these 31 RBPs, 24 RBPs were reported from RNAcompete study only, two RBPs were reported through SELEX and yeast three-hybrid screening, and five RBPs were reported from RNAcompete and SELEX/RIP-Chip. These motifs were also searched in a similar manner. Also, TOMTOM was applied to evaluate the significance of similarity observed between the experimentally reported motif and the corresponding matching motif from RBPSpot.

#### Application of RBPSpot across SARS-CoV2 genome

To identify the binding sites of RBPs across SARS-Cov2 genome, we downloaded its genome from NCBI (accession number NC_045512).

### Quantification and statistical analysis

#### Statistical analysis

K-mers and motifs enrichment analysis: A binomial test was done for significant motif enrichment analysis between crosslinking and random data. Null model probabilities were derived using the randomly selected regions from RNAs/regions not reflected in the CLIP-seq data. All those k-mers which qualified the test at significance level cutoff of 1% were considered for the further analysis as significant ones. Statistical test was performed using binom.test(x, n,p) function in R standard package. Here, ‘x’ represents the total number of peak data instances with the prime motif, ‘n’ is the total number of instances for any given RBP, and ‘p’ is the probability of occurrence of the prime motif in the random data.

Co-occurring motifs analysis: A KS-test was performed for the co-occurrence rate for the motif pairs for any given distance. Motif pairs’ co-occurrence was calculated for -75 to +75 bases distance in the random and crosslinking data. The distribution of co-occurrence count at each position for random and crosslinking data were considered as ‘x’ and ‘y’ variables in ks.test(x,y) function of R standard package. The p-value cutoff of 0.01 was considered to screen the significantly co-occurring motif pairs.

Features’ discriminative power evaluation: A Student t-test was performed between the positive and negative instances for the dinucleotides, pentameric and heptameric features for the highest F-score valued positions. The probability values for pentamers, heptamers, and densities values for dinucleotides for the highest F-score value positions between positive and negative instances were used as the ‘x’ and ‘y’ variables in t.test(x,y) function of R standard package. The p-value cutoff of 0.01 was considered as the significant one to distinguish between positive and negative instances probabilities score.

Benchmarking performance significance evaluation tests: A series of Student’s t-tests was conducted for benchmarking performance significance evaluation across the three different datasets for the compared six tools, including RBPSpot. In this analysis, the five different tools were compared with RBPSpot one by one on the basis of performance metrics (MCC/Accuracy scores). A SciPy python function scipy.stats.ttest_ind(x,y) was used for this part of the analysis, where ‘x’ and ‘y’ represent the considered metric score (MCC/Accuracy) for all the studied RBPs for RBPspot and for the software being compared to RBPSpot, respectively. Significance cut-off p-value of 0.01 was applied. Same approach was applied when RBPSpot, DeepCLIP, and iDeepE were compared for different combinations of training and testing datasets in the latter part of the benchmarking analysis.

## Data Availability

• All the secondary data used in this study were publicly available and their due references and sources have been provided in Table S13. Dataset resource link is provided in Key resource table. • The software has been made available at Github at:https://github.com/SCBB-LAB/RBPSpot and https://scbb.ihbt.res.in/RBPSpot/. • All other data and information generated/used, methodology related details etc have also been made available in the supplementary data files provided along with and also made available through the related open access server at https://scbb.ihbt.res.in/RBPSpot/.

## Supplemental information

Document S1. Figures S1–S3 and Table S2

Table S1. Primary motif for each RBP and percentage of it's occurrence, related to Figure 11

Table S3. Co-occurring motifs information, their sequence and percentage of co-occurrence, related to Figure 1

Table S4. Top 100 features for each RBP on the basis of f-score, related to Figure 3

Table S5. Machine learning statistics for all the RBP of set A, related to Figure 5

Table S6. Machine learning statistics for all the RBP of set, related to Figure 5

Table S7. Benchmarking evaluation for RBPSpot on different composition of dataset, related to Figure 8

Table S8. Benchmarking evaluation for iDeepE on different composition of dataset, related to Figure 8

Table S9. Benchmarking evaluation for DeepCLIP on different composition of dataset, related to Figure 8

Table S10. Benchmarking evaluation for different tools on beRBP dataset, related to Figure 7

Table S11. Benchmarking evaluation for different tools on RBPSpot dataset, related to Figure 7

Table S12. Benchmarking evaluation for different tools on Graphprot dataset, related to Figure 7

Table S13. Primary data information including accession id, tissue and study, related to Figure 11

Table S14. Initial peak data download from ENCORI and final Peak data after filtering peak length > 5bp to ≤300 Bp, related to Figure 11

Table S15. Finalized hyperparameter detail used for all 131 RBP, related to Figure 4

Table S16. 10-Fold cross validation stats for all 131 RBPs, related to Figure 5
