# Supplementary material for: RBPSpot: Learning on appropriate contextual information for RBP binding sites discovery
Source: iScience. 2021 Oct 30;24(12):103381. doi: 10.1016/j.isci.2021.103381 (PMC8605353; doi:10.1016/j.isci.2021.103381)
Supplement: Document S1. Figures S1–S3 and Table S2 [file mmc1.pdf]

iScience, Volume *24*

Supplemental information

RBPSpot: Learning on appropriate contextual information for RBP binding sites discovery

Nitesh Kumar Sharma, Sagar Gupta, Ashwani Kumar, Prakash Kumar, Upendra Kumar Pradhan, and Ravi Shankar

Supplementary Figure:

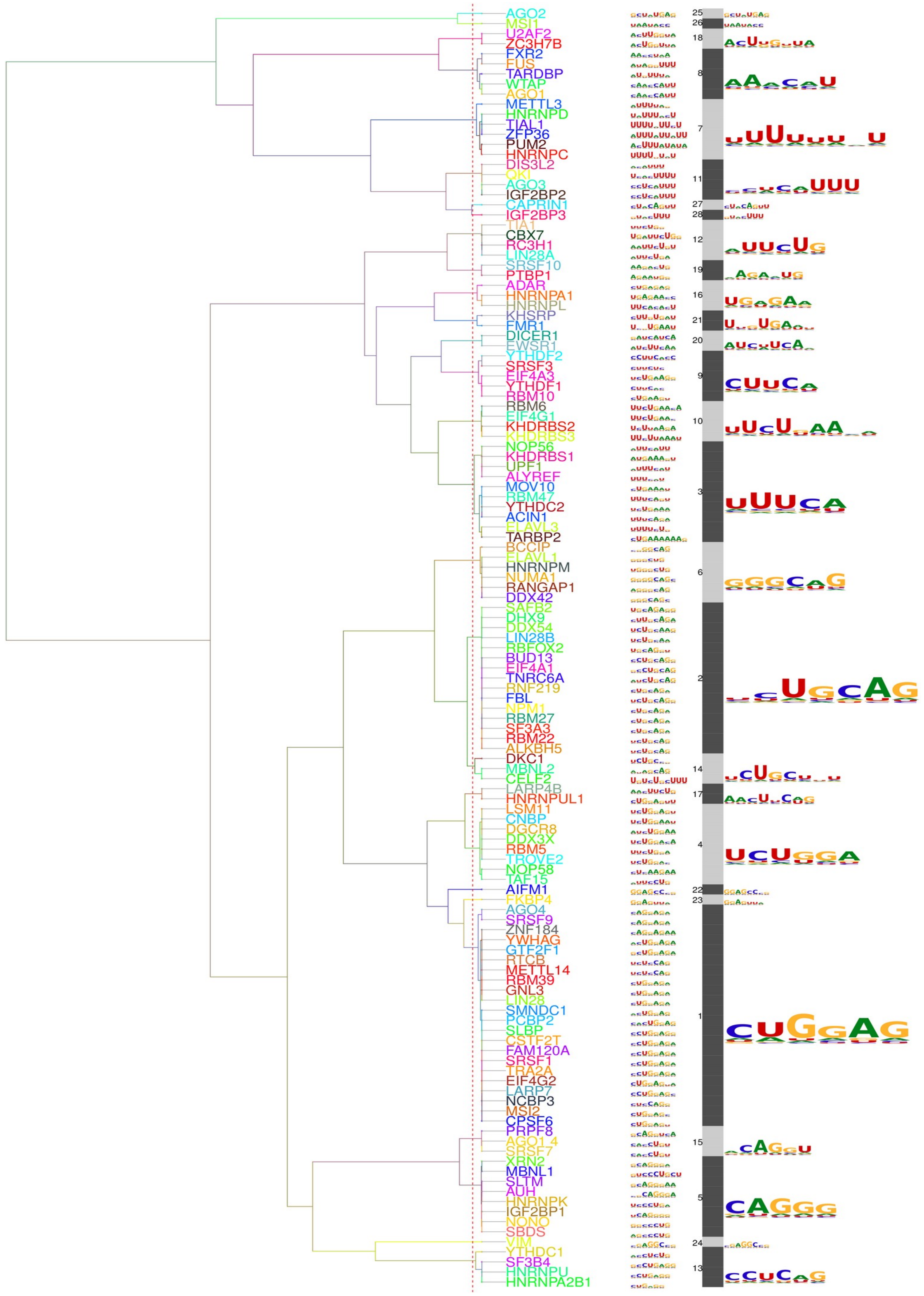

FigureS1: Motif clustering of the RBPs. A total of 28 different clusters for 127 different RBP were formed. The members of these clusters shared high similarity among their binding site prime motifs. However, despite of sharing close similarity, their binding were found highly contextual. **Related to Figure 1.**

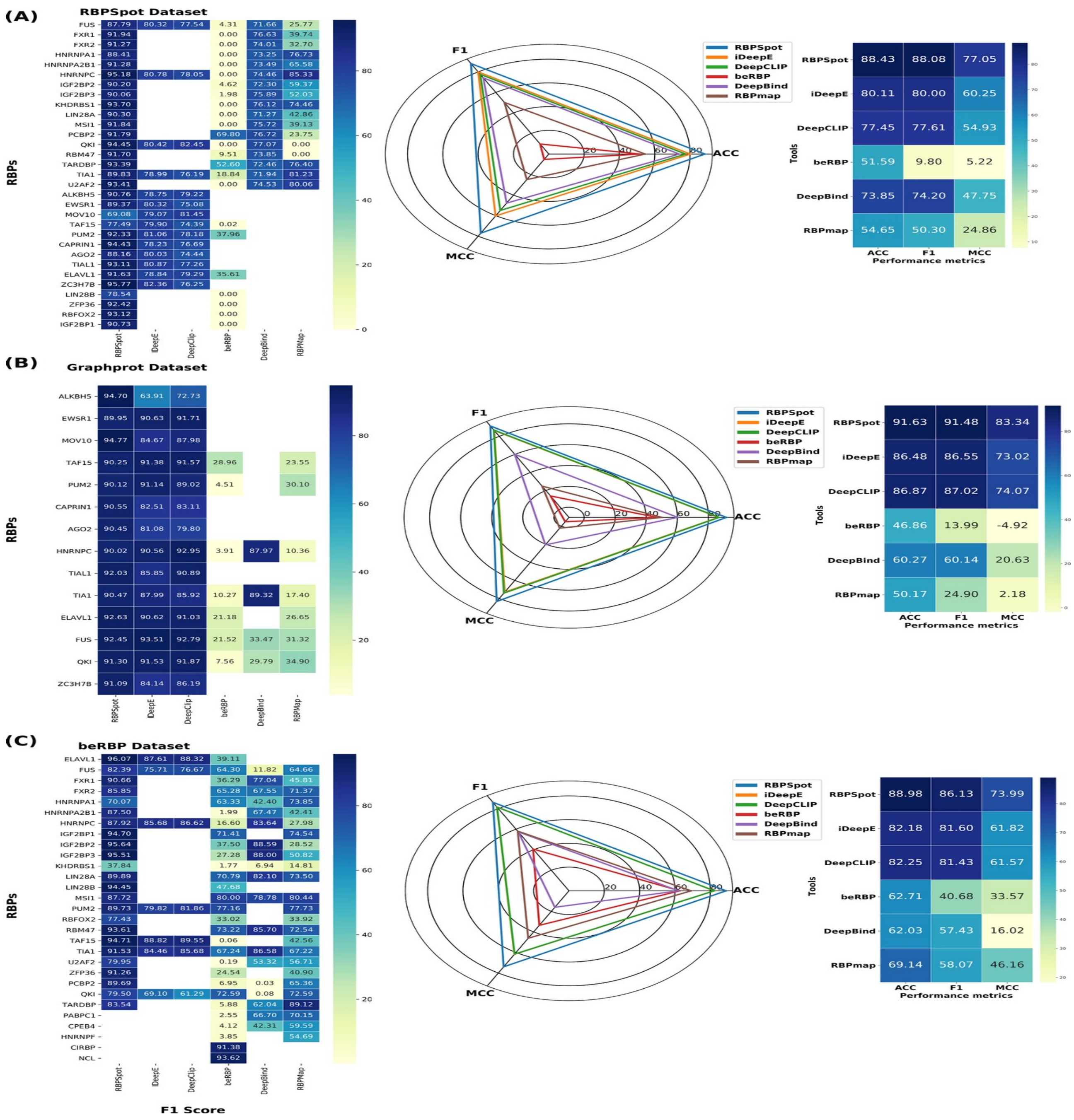

FigureS2: Comparative bench-marking results of RBPSpot when compared to beRBP, DeepBind, RBPmap, iDeepE, and DeepCLIP for three different datasets. (A) Bechmarking result on RBPSpot dataset, (B) Graphprot dataset, and (C) beRBP dataset. Each of these datasets performances was evaluated for various performance metrics where the heatmaps are for F1-Score. Radar charts view of the average Accuracy, F1 score, and MCC attained by each software for the corresponding dataset. Rightmost heatmap showing the average accuracy, F1-score and MCC for the same. **Related to Figure 7.**

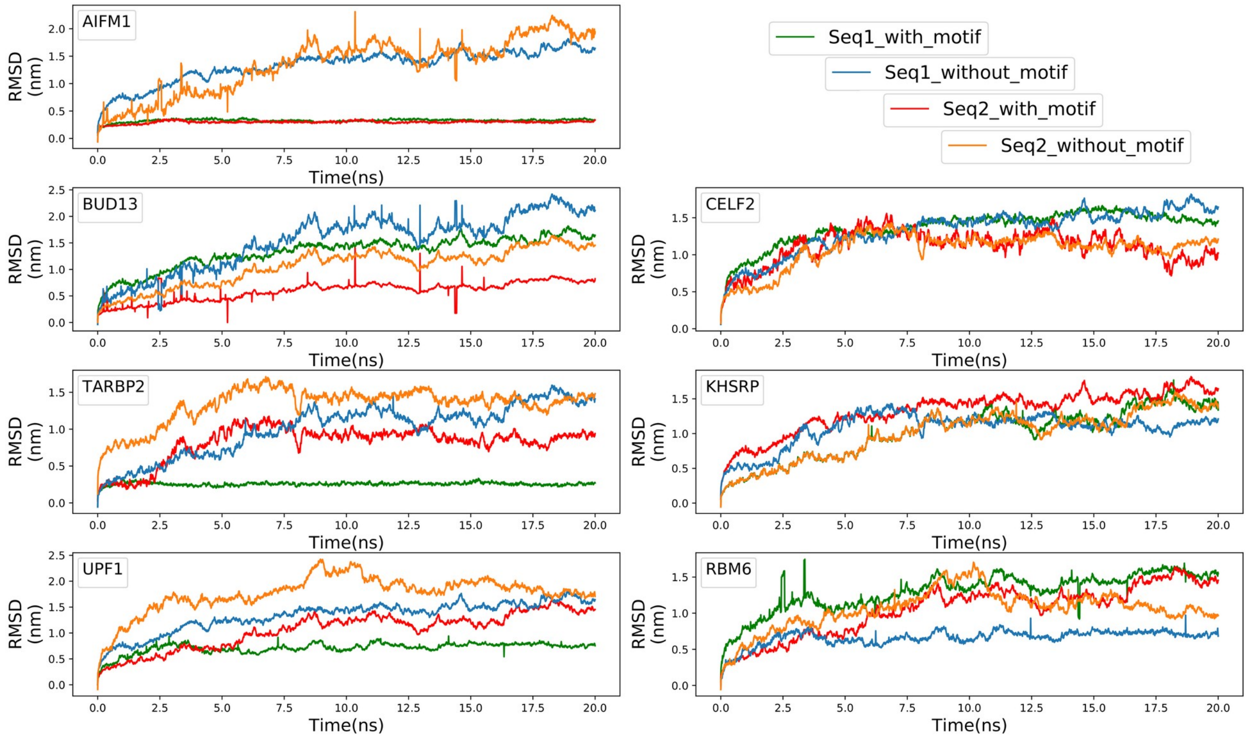

FigureS3: Comparative time dependent root mean square deviations (RMSD) plots for the seven different RBP-RNA complexes for interaction with nSARS-CoV-2 genome. It was measured with and without the main motif. The trajectory was measured at 300 K for the 20-ns. The molecular dynamics study supported most of the discovered binding sites identified by RBPSpot. **Related to Figure 10.**

Table S2: RBPs with mutually exclusive motifs. **Related to Figure 11**.

RBP Name	Primary Motif (Motif 1 / Motif 2)	Individual motif occurrence in Peaks	Motif occurrence in Peaks	Total number of peak data	Percentage of data
FXR1	UGAAUA+CUGGAG	47868+55357	99457	107568	92.46
SND1	ACUUUA+GGGUCA	69452+87227	151918	169428	89.67
ILF3	UGGACG+UUGGUA	32011+25121	54894	64030	85.73
U2AF1	UCUUCU+ACCUGU	111661+83892	187574	210520	89.1
